# A hidden intrinsic ability of bicistronic expression based on a novel translation reinitiation mechanism in yeast

**DOI:** 10.1093/nar/gkaf220

**Published:** 2025-03-28

**Authors:** Yiwen Sun, Ralph Bock, Zhichao Li

**Affiliations:** Key Laboratory of Systems Microbial Biotechnology, Tianjin Institute of Industrial Biotechnology, Chinese Academy of Sciences, Tianjin 300308, China; University of Chinese Academy of Sciences, Beijing 100049, China; National Technology Innovation Center of Synthetic Biology, Tianjin 300308, China; Max Planck Institute of Molecular Plant Physiology, Am Mühlenberg 1, 14476 Potsdam-Golm, Germany; Key Laboratory of Systems Microbial Biotechnology, Tianjin Institute of Industrial Biotechnology, Chinese Academy of Sciences, Tianjin 300308, China; University of Chinese Academy of Sciences, Beijing 100049, China; National Technology Innovation Center of Synthetic Biology, Tianjin 300308, China

## Abstract

Gene organization in operons and co-expression as polycistronic transcripts is characteristic of prokaryotes. With the evolution of the eukaryotic translation machinery, operon structure and expression of polycistrons were largely abandoned. Whether eukaryotes still possess the ability to express polycistrons, and how they functionally activate bacterial operons acquired by horizontal DNA transfer is unknown. Here, we demonstrate that a polycistron can be rapidly activated in yeast by induction of bicistronic expression under selection. We show that induced translation of the downstream cistron in a bicistronic transcript is based on a novel type of reinitiation mediated by the 80S ribosome and triggered by inefficient stop codon recognition, and that induced bicistronic expression is stable and independent of *cis-*elements. These results provide key insights into the epigenetic mechanism of the pathway of activation. We also developed a yeast strain that efficiently expresses bicistronic constructs, but does not carry any genomic DNA sequence change, and utilized this strain to synthesize a high-value metabolite from a bicistronic expression construct. Together, our results reveal the capacity of yeast to express bicistrons in a previously unrecognized pathway. While this capacity is normally hidden, it can be rapidly induced by selection to improve fitness.

## Graphical Abstract

**Graphical Abstract:**
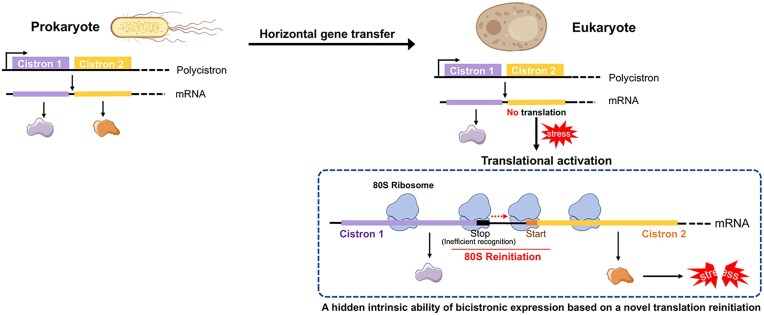

## Introduction

In prokaryotes, genes are commonly arranged in operons that allow the concerted expression of functionally related genes as polycistronic transcripts, thus enabling the efficient regulation of complex biological process [1–3]. As eukaryotes evolved 5′ mRNA cap-dependent translation initiation mechanisms, operon structure and expression of polycistrons were largely abandoned. Due to their bacterial origin, mitochondria and plastids (chloroplasts) also retained the mechanisms for co-expression of multiple genes from polycistronic transcripts [4]. Polycistronic mRNAs are rare in eukaryotes, but have been observed in nematodes [5, 6] and tunicates [7–9], where they undergo trans-splicing to process the polycistronic precursor RNA into mature monocistronic messenger RNAs (mRNAs). In addition, bicistronic and polycistronic transcripts have been discovered in two species of green algae that appear to translate them by leaky ribosome scanning [4]. Moreover, some eukaryotic genes harbor short upstream open reading frames (uORFs, each encoding only a few amino acids), and the main reading frame is translated via reinitiation (REI based on uORFs) mediated by the 40S ribosomal subunit, thus also resembling a polycistronic expression mechanism [10–12]. In yeast, REI can also occur if there are insufficient amounts of a ribosome dissociation factor (such as Rli1) in the cell [13]. This limits the assembly of complete termination/pre-recycling complexes (composed of the ribosome-nascent chain complex, release factors and ribosome dissociation factors) upon recognition of the termination signal (stop codon) [14–16]. As a result, some 80S ribosomes are not dissociated and can undergo REI, albeit typically leading only to low expression of short ORFs in the 3′ untranslated region (UTR). This 80S-REI is analogous to the 70S-scanning initiation in prokaryotes. Shine-Dalgarno (SD) sequence is important for ribosomal landing in the 70S-scanning process, while the eukaryotic 80S-REI does not necessitate such an element [17]. However, neither of the two known REI mechanisms of eukaryotes can mediate bicistronic or polycistronic expression. As eukaryotes are believed to generally lack the ability to express polycistronic mRNAs, several artificial tools of multigene expression from a single transcript for eukaryotes (represented by yeast) were developed mainly based on viral elements. The tools include the bicistronic strategy and polyprotein synthesis from one translation unit (reading frame). The bicistronic approaches rely on the exogenous elements, such as internal ribosomal entry sites (IRES) [18–20] and intergenic sequence 6-mediated REI (IGG6) [21], while 2A peptides can generate two independent peptides from a single ORF via a stop-start mechanism [11, 18, 22, 23].

Polycistronic transcription units can be transferred from bacteria to eukaryotic recipients via horizontal gene transfer (HGT) [2, 24–28], defined as any movement of genetic material between organisms other than by descent. However, in none of these examples, the original polycistronic (operon) structure has been retained. Instead, the genes were rearranged and are now expressed as individual monocistronic units. Genes horizontally transferred from bacteria to eukaryotes are expected to be initially silent in the recipient organism because of the different gene expression systems and, especially, the inability of eukaryotes to faithfully express transferred polycistrons. Rapid functional activation of the transferred genes is necessary to confer a fitness advantage and prevent the evolutionary deterioration by accumulation of mutations [2, 29, 30]. The mechanism leading to the rapid activation of horizontally transferred bacterial operons in eukaryotic recipients are still largely unknown. There are multiple barriers to functional HGT posed by incompatibilities in the transcription and translation mechanisms [29, 31, 32], including promoter recognition, inability to translate a polycistronic mRNA, fundamentally different mechanisms of translation initiation, and differences in codon usage [2, 29, 33]. Previous studies have shown that codon usage requirements are not strict [30, 34], and promoter incompatibility can be relatively easily overcome by promoter acquisition at the insertion site or promoter capture from adjacent gene [30, 31]. The captured promoter will usually also provide suitable translation initiation signals. By contrast, the mechanism(s) by which eukaryotic cells overcome the barrier of polycistronic gene expression remains elusive [2].

To gain insight into whether eukaryotes still possess the ability to express polycistrons and how they functionally activate bacterial operons acquired by horizontal DNA transfer, we have inserted a prokaryotic-type polycistron consisting of three yeast selectable marker genes into the genome of *Saccharomyces cerevisiae*. We found that the polycistronic structure indeed prevented expression of the two downstream genes (cistron 2 and cistron 3), although the complete polycistron could be efficiently transcribed. When subsequent selection for functional activation of the polycistron was conducted, we observed that the translation of cistron 2 was induced unexpectedly rapidly, whereas the activation of cistron 3 depended on the ability to translate cistron 2, and additionally required genomic rearrangements. We also report that the induced bicistronic expression competence was independent of any *cis-*element and persists for many rounds of subculture in the absence of selection. Our data suggest that cistron 2 is efficiently translated based on a new type of REI that is mediated by the 80S-ribosome and evoked by inefficient stop codon recognition. Surprisingly, the observed REI induction was independent of genomic mutations but likely involves epigenetic modifications and downregulation of *NDJ1*, a gene encoding a telomere-associated meiotic protein. Finally, we developed a yeast strain with the capacity for bicistronic expression (which does not harbor any changes in the genome), and we demonstrated the utility of this strain by synthesizing a high-value metabolite (provitamin A) from a bicistronic expression construct. Hence, we reveal the hidden intrinsic ability of bicistronic expression based on a novel type of translation reinitiation in yeast and develop a novel and efficient gene expression technology and make yeast even more valuable for basic research and industry.

## Materials and methods

### Construction of vectors and strains

*S. cerevisiae* strains were cultured at 30°C on YPD (1% yeast extract, 2% peptone, and 2% glucose) or SD (0.67% yeast nitrogen base without amino acids, 2% glucose) supplemented with appropriate amino acids and bases, as previously described [35]. For prion elimination, strains were treated with five rounds of growth on YPD agar plates containing 4 mM guanidine hydrochloride (GdnHCl) [36]. Low copy (lc) and high copy (hc) shuttle plasmids are based on pRS415 and pRS425, respectively. The *LEU2* gene of pRS415 and pRS425 was replaced with the G418 resistance gene to form pRS415G and pRS425G. The substitutions of marker genes were introduced into the various plasmids by fusion polymerase chain reaction (PCR), and the plasmids were constructed by Gibson cloning using the Minerva Super Fusion Cloning Kit (UElandy, Suzhou, China). The hairpin sequence utilized in this study is 5′-AGATCTGGTACCGAGCTCCCCGGGCTGCAGGATATCCTGCAGCCCGGGGACCTCGGTACCAGATCT-3′.*lacZ* used in lcGCN4&L:G was amplified from the wild-type *Escherichia coli* genome. *GGPPS* (UniProtKB accession number Q9P885), *CARB* (UniProtKB accession number Q67GI0), and *CARRP* (UniProtKB accession number Q9UUQ6) of the β-carotene pathway were codon-optimized for expression in *S. cerevisiae* and synthesized by Genewiz (Suzhou, China). Yeast transformations were conducted by the standard lithium acetate method [37].

The *E. coli*-type polycistron was constructed by combining three selectable marker genes with the intercistronic region of the *lac* operon via PCR, and the resulting fragment was integrated downstream of the promoter of the *FAA1* gene in the BY4741 genome to form the original strain BY4741-L^A^:U:H. The endogenous gene *URA3* was deleted from BY4741-L^A^:U^A^:H by homologous recombination, and the homologous arms were located on the flanks of *URA3* in the L:U:H polycistron, resulting in BY4741-L^A^:U:H △*URA3*. The other genome-edited strains were constructed using the CRISPR–Cas9 system, in which sgRNA fragments were formed through annealing of two complementary oligonucleotides. d*NDJ1* refers to the knock-down of the expression level of *NDJ1* using the CRISPR/dCas9 (D10A and H840A)-Mxi1 system in BY4741-L^A^:U:H.

### Selection for functional activation of the prokaryotic-type polycistron in yeast

A genetic screen for functional activation of the polycistron was conducted by exposing the original strain in the auxotroph medium. Fifty milliliters of auxotroph medium inoculated with the original strain at OD_600_ of ∼0.1 was cultivated at 30°C and 180 rpm.

### Stability assay for bicistronic expression

The assay was based on 20 successive subcultures of strains at a 1:50 dilution in complete medium. Subculturing was performed every 24 h, and sample from each subculture was collected for fluorescence analysis.

### Nucleic acid isolation, gel blot analysis, 5′ rapid amplification of cDNA ends, and quantitative reverse-transcription PCR

Yeast total RNA was extracted with the Yeast RNA Kit (Omega, GA, USA), and DNA was removed using RNase-free DNase I (Omega, GA, USA). Total DNA was isolated with the Yeast Genomic DNA Extraction Kit (Solarbio, Beijing, China).

For northern blot analysis, samples of 10 μg total RNA were separated in denaturing formaldehyde-containing agarose gels (1.3%) and transferred onto Hybond XL nylon membranes (GE Healthcare, Buckinghamshire, UK) by capillary blotting. For Southern blot analysis, DNA samples (∼5 μg) were digested with two different restriction enzymes, SacI and ApaLI, for 16 h, separated by electrophoresis in 1% agarose gels and transferred onto nylon membranes. Labeling of the gene-specific probes (200 bp) and hybridization were performed with the DIG-High Prime DNA Labeling and Detection Starter Kit II following the manufacturer’s instructions (Roche, Indianapolis, IN, USA). The membrane was exposed to a chemiluminescence reagent and exposed to a luminescence imager (Tanon 5200 Multi, Shanghai, China).

5′ rapid amplification of cDNA ends (5′ RACE) was performed using the 5′ RACE Kit (Tianjingsha, Beijing, China). For quantitative reverse-transcription PCR (qRT-PCR) analysis, first-strand complementary DNA (cDNA) was synthesized from total RNA using M-MLV reverse transcriptase (Promega, Madison, WI, USA) and oligo-dT primer. Subsequent amplification was performed using the LightCycler 96 Real-Time PCR System (Roche) and the ChamQ Universal SYBR qPCR Master Mix (Vazyme Biotech, Nanjing, China) with *ACT1* as a reference gene. Three biological replicates were analyzed with three technical replicates each. The 2^−△△CT^ (cycle threshold) method was used to determine relative cDNA levels. Oligonucleotides used are listed in Supplementary Table S2.

### Protein extraction and immunoblot analysis

Total protein was extracted with the Yeast Total Protein Extraction Mini Kit (Coolaber, Beijing, China) and quantified using the Bradford method. Total protein samples were denatured at 100°C for 5 min in sample buffer (50 mM Tris–HCl, pH 6.8, 2% SDS, 0.1% bromophenol blue, 10% glycerol, and 1% β-mercaptoethanol), separated in denaturing 12% sodium dodecyl sulfate polyacrylamide (SDS–PAA) gels and transferred onto polyvinylidene difluoride membranes (Hybond^™^ P; GE Healthcare) in a standard Tris-glycine transfer buffer (25 mM Tris–HCl, 192 mM glycine, and 20% methanol). Immunochemical protein detection was performed using a 1:5000 dilution of monoclonal antibody. The primary antibodies used were anti-GFP (HT801, TransGen Biotech, Beijing, China), anti-FLAG (HT201, TransGen), anti-β-Actin (ab170325, Abcam, Cambridge, UK), and a 1:5000 dilution of anti-mouse HRP-conjugated secondary antibody (AS003, Abclonal, Wuhan, China). Hybridization signals were visualized by Tanon^™^ Femto-sig ECL and recorded in a luminescence imager (Tanon 5200 Multi, Shanghai, China).

### Fluorescence and β-galactosidase activity assays

The strains carrying the fluorescent protein gene were cultured overnight, then resuspended to an absorbance at 600 nm of 0.1, and grown at 30°C for 18 h. Subsequently, 0.2 ml of yeast cells were harvested by centrifugation at 4000 × *g* for 2 min and diluted to an optical density at 600 nm of 0.5 with phosphate-buffered saline. Fluorescence was measured using the Synergy Neo2 instrument (BioTek, Vermont, USA). GFP fluorescence was assessed at excitation wavelength of 483 nm and emission wavelength of 520 nm, while RFP fluorescence was measured at 585–620 nm. Relative fluorescence intensity was derived as fluorescence/optical density at 600 nm.

The β-galactosidase (β-GAL) activity was determined with the β-galactosidase Activity Assay Kit (Solarbio, Beijing, China).

### Yeast two-hybrid assay and protein-protein binding simulations

Yeast two-hybrid (Y2H) assays were performed using the Matchmaker Yeast Two-Hybrid System (Clontech Laboratories, Mountain View, CA, USA), according to the manufacturer’s instructions. Briefly, specific combinations of the AD and BD vectors were used to co-transform yeast cells, followed by selection on -Leu/-Trp DO (DDO; double dropout) or -Leu/-Trp/-Ade/-His DO (QDO; quadruple dropout) medium plates at 30°C. Yeast cells co-transformed with pGADT7-T and pGBKT7-53 (pAD-T and pBD-53) were used as a positive control, and yeast cells co-transformed with pGBKT7 (BD) were used as a negative control.

### Whole-genome resequencing analysis

High-quality DNA samples were shipped on dry ice to Genewiz (Suzhou, China) for DNA library construction and next-generation sequencing. For each sample, 0.2 mg of genomic DNA was randomly fragmented (by Covaris) to an average size of 300–350 bp. The fragments were treated with End Prep Enzyme Mix for end repair, 5′ phosphorylated, and 3′ adenylated to add adaptors to both ends. Size selection of adaptor–ligated DNA was then performed by DNA Cleanup beads. Each sample was then amplified by PCR for 8 cycles using P5 and P7 primers, with both primers carrying sequences which can anneal with flowcell to perform bridge PCR and the P7 primer carrying a six-base index allowing for multiplexing. The PCR products were cleaned up and validated using an Agilent 2100 Bioanalyzer. The qualified libraries were paired-end sequenced (PE150) on the Illumina Novaseq System. The fastp (V0.23.0) tool was used to remove the sequcences of adaptors, PCR primers, N base >14, and Q20 <40%. The Sentieon (V202112.02) pipeline was used to map clean data to the reference genome, remove duplications, and call SNVs/InDels.

### Transcriptome analysis

High-quality RNA samples were shipped on dry ice to Biomarker Biotechnology Corporation (Beijing, China) for cDNA library construction and RNA sequencing. Each strain served as one transcriptomic sample, and each sample was analyzed in three biological replicates. The library was prepared using the VAHTS^®^ Universal V8 RNA-seq Library Prep Kit for Illumina (Vazyme). The cDNA libraries were then paired-end sequenced on an Illumina NovaSeq 6000 platform (Illumina, San Diego, CA, USA). Clean reads were derived by eliminating sequences containing adapters, poly-N, and low-quality reads from the raw data. Simultaneously, the percentage of Q30 bases and the GC content of the cleaned data were computed. All subsequent analyses were conducted exclusively on this set of high-quality clean data. The high-quality reads were subsequently mapped to the reference genome of *S. cerevisiae* strain S288C (GCF_000146045.2_R64) using HISAT2, and the mapped reads were subsequently assembled using StringTie. Gene expression levels were calculated based on the read counts and normalized by the fragments per kilobase of transcript per million fragments mapped (FPKM) method. Differentially expressed genes (DEGs) were identified using the DESeq2 package using a false discovery rate (FDR) < 0.01 and fold-change (FC) ≥ 2 as the filtering criteria.

### Quantitative analysis of nucleic acid modifications

Nucleic acid modifications were detected by LC-MS/MS performed by CloudSeq Inc. (Shanghai, China). For DNA methylation modification, samples of 4 μg DNA were digested with restriction enzymes BseR **I** and then denatured at 95°C for 5 min. For RNA modification, 1 μg of RNA sample was mixed with a buffer containing S1 nuclease, phosphodiesterase, and alkaline phosphatase, and incubated at 37°C to completely hydrolyze RNA into nucleosides. The processed sample was mixed with a buffer containing S1 nuclease, alkaline phosphatase, and DNase, and incubated at 37°C to completely hydrolyze the DNA into nucleosides. Subsequently, the sample (DNA or RNA) was extracted with chloroform, and the upper aqueous solution was collected. The nucleosides were detected using an AB Sciex QTRAP 6500 LC-MS/MS platform in positive ion mode. Quantification was performed by comparison against a standard curve obtained from pure nucleoside standards running in the same batch.

## Results

### Yeast cannot normally translate polycistronic transcripts

**Figure 1.**
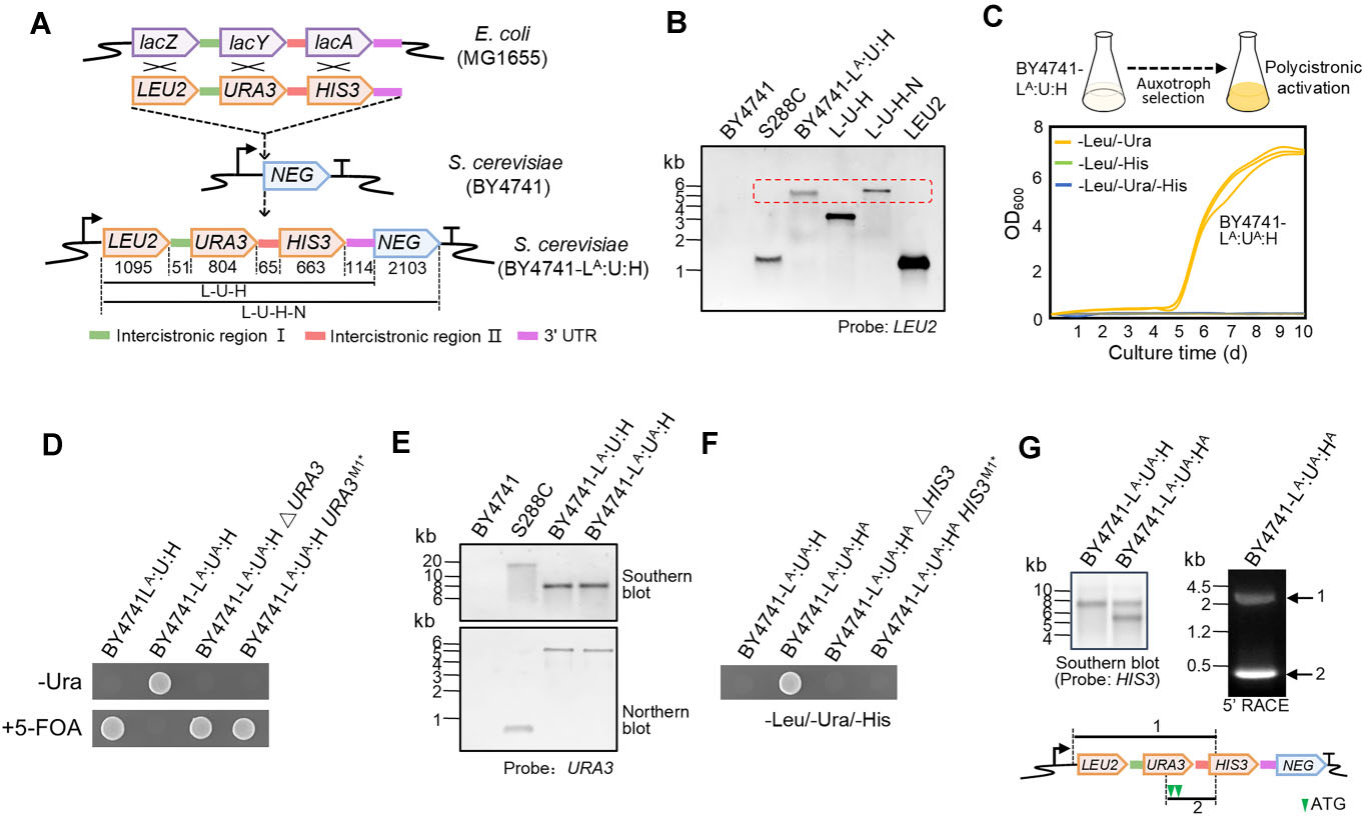
Rapid functional activation of a prokaryotic-type polycistron in yeast. (**A**) Schematic map of the model operon used to mimic a horizontally transferred prokaryotic-type polycistron in *S. cerevisiae*. The three reading frames of the *lac* operon were replaced by three yeast selectable markers (*LEU2*, *URA3*, and *HIS3*), and the resulting *E. coli*-type polycistron was inserted downstream of the promoter of a non-essential gene in the genome of *S. cerevisiae* (BY4741), mimicking horizontal operon transfer from prokaryote to eukaryote with promoter acquisition. Numbers indicate the length (base pairs) of the corresponding regions. *NEG*, non-essential gene *FAA1*; bent arrow, promoter of *FAA1*; superscript A, gene function is active. (**B**) The transferred polycistron is transcribed into a full-length mRNA, as evidenced by northern blot analysis with a *LEU2*-specific probe. In the first three lanes, total RNA from BY4741, S288C, and BY4741-L^A^:U:H was loaded. BY4741 is a negative control that lacks *LEU2* in its genome. S288C is a wild-type yeast strain with *LEU2* in the genome. The last three lanes show PCR products of L-U-H, L-U-H-N, and *LEU2* generated from genomic DNA of BY4741-L^A^:U:H as template. The dotted rectangle indicates the expected size of the full-length transcript. (**C**) Functionality of cistron 2 is rapidly activated upon selection. Selection for functional activation of the polycistron was conducted by incubating BY4741-L^A^:U:H in dropout media. -Leu/-Ura medium was used to select for functional activation of cistron 2, -Leu/-His medium for activation of cistron 3, and -Leu/-Ura/-His medium for activation of both cistrons. The activation process is monitored by growth curves, each selection experiment was conducted in three biological replicates. Only cistron 2 could be functionally activated within several days, and the activated strain was named BY4741-L^A^:U^A^:H. (**D**) Functionality of cistron 2 is acquired by activation of its expression. In strain BY4741-L^A^:U^A^:H △*URA3*, the *URA3* reading frame of the L:U:H polycistron was knocked out from BY4741-L^A^:U^A^:H by homologous recombination. In strain BY4741-L^A^:U^A^:H △URA3^M1*^, the start codon AUG of *URA3* of the L:U:H polycistron was mutated to a stop codon. Strains were cultured on medium without uracil or supplemented with 5-fluoroorotic acid (5-FOA) to enable positive or negative selection for *URA3* expression. (**E**) Functional activation is achieved by translation of cistron 2 from the polycistronic transcript. Southern blot analysis (upper panel) was performed to test for genomic rearrangements. Total DNA of the yeast strains indicated was digested with the restriction enzymes SacI and ApaLI, which cut outside of the polycistron. Detection of the expected restriction fragment of 7011 bp suggests unaltered genomic localization and structure of the operon (further confirmed by DNA resequencing). Northern blot analysis (lower panel) was performed to test for possible alterations in transcript patterns. Samples of total RNA were electrophoretically separated in a 1.3% denaturing agarose gel, blotted and hybridized to a digoxigenin-labeled probe specific for the *URA3* coding region. BY4741 is a negative control that lacks *URA3* in its genome. S288C is a wild-type yeast strain with *URA3* in the genome. (**F**) Strain BY4741-L^A^:U^A^:H evolved a functional cistron 3. Strain BY4741-L^A^:U^A^:H^A^ evolved from strain BY4741-L^A^:U^A^:H after approximate half a month of selection on -Leu/-Ura/-His medium, while the original strain BY4741-L^A^:U:H could not evolve histidine prototrophy. Deletion and nonsense mutation (start codon to stop codon) of cistron 3 (*HIS3*) from BY4741-L^A^:U^A^:H^A^ abrogated the acquired histidine prototrophy, confirming functional activation of *HIS3* in BY4741-L^A^:U^A^:H^A^. (**G**) The functional activation of cistron 3 is caused by a gene duplication event of cistrons 2 and 3. Southern blot analysis indicates presence of an additional copy of *HIS3* in the genome of BY4741-L^A^:U^A^:H^A^. 5′ RACE revealed an additional shorter-than-expected *HIS3* transcript. Sequence analysis showed that this additional transcript does not contain cistron 1 (*LEU2*) but includes a part of cistron 2 (*URA3*) that harbors several in-frame start codons that could be utilized for translation initiation, as indicated in the schematic gene map (bottom panel).

The transcriptional barrier of HGT can be easily overcome by promoter acquisition [30], but the eukaryotic translation mechanism will prevent the expression of functional proteins from a polycistronic mRNA. To elucidate the mechanisms that lead to functional activation of operon genes, the three genes of the *E. coli lac* operon were replaced by three yeast selectable markers (*LEU2*, *URA3*, and *HIS3*) to facilitate the detection of polycistronic gene expression. The resulting *E. coli*-type polycistron was inserted downstream of the promoter of a non-essential gene in the genome of *S. cerevisiae* BY4741 (Fig. 1A), thus mimicking horizontal operon transfer from a prokaryote to a eukaryote and subsequent promoter acquisition. Northern blot analysis revealed the presence of full-length transcripts (Fig. 1B), indicating that the polycistronic structure did not hinder transcription in yeast. As expected, the transferred polycistron enabled yeast to grow on -Leu medium, indicating that cistron 1 was faithfully translated. However, the strain was unable to grow on -Ura or -His medium, indicating that cistrons 2 and 3 could not be translated into functional proteins. Accordingly, this strain was named BY4741-L^A^:U:H.

### Rapid functional activation of a prokaryotic-type polycistron in yeast

To investigate how the translational barrier caused by the polycistronic structure can be overcome in evolution, selection for functional activation of the polycistron was conducted by growing BY4741-L^A^:U:H cultures in dropout media (Fig. 1C, upper panel). All three biological replicates of BY4741-L^A^:U:H started to grow in uracil dropout medium after ∼5 days, and these cultures were tentatively treated as the cistron 2-activated strain BY4741-L^A^:U^A^:H (Fig. 1C, bottom panel). The high repeatability of the activation process suggested that cistron 2 expression may be an inherent (and possibly inducible) property of yeast cells. However, strain BY4741-L^A^:U:H could not grow in histidine dropout medium even after prolonged cultivation times, suggesting that the expression of cistron 3 cannot be readily induced in our experimental design. To verify that growth was indeed due to *URA3* expression rather than adaptive evolution, we knocked out *URA3* (or mutated its start codon to a stop codon) from the L:U:H polycistron in BY4741-L^A^:U^A^:H. The resulting strains could not grow on uracil dropout medium (Fig. 1D).

In theory, *URA3* activation could be caused by acquisition of a separate promoter for monocistronic expression. However, Southern blot analysis revealed that the activated strain possessed no additional copy of *URA3*, and northern blot experiments showed that the *URA3* transcript had the same size as in the original strain (Fig. 1E). Moreover, DNA sequencing confirmed that there were no mutations in the polycistronic cassette in the activated strain. These results indicated that the activation of *URA3* was due to cistron 2 translation in the activated strain.

Because it was not possible to activate cistron 3 in strain BY4741-L^A^:U:H (Fig. 1C), we plated strain BY4741-L^A^:U^A^:H (with activated cistron 2 translation) onto solid -Leu/-Ura/-His medium to screen for histidine auxotrophy at large scale and facilitate the efficient isolation of cistron 3 activation events as single colonies [29, 30]. As a result, we isolated one colony (referred to as BY4741-L^A^:U^A^:H^A^), which could grow on triple-dropout medium (Fig. 1F). Deletion and nonsense mutation of *HIS3* from BY4741-L^A^:U^A^:H^A^ abrogated the growth phenotype, confirming the functional activation of *HIS3* in this strain (Fig. 1F). In contrast to the rapid and highly reproducible induction of growth of BY4741-L^A^:U^A^:H, the isolation of the only BY4741-L^A^:U^A^:H^A^ event required a long screening time of half a month, possibly suggesting that the *HIS3* activation event may be attributable to a *de novo* mutation (e.g. a genomic rearrangement). Indeed, Southern blot analysis detected an additional copy of *HIS3* (Fig. 1G, left panel). 5′ RACE analysis also revealed an additional shorter *HIS3*-containing transcript (Fig. 1G, right). cDNA sequencing showed that this additional transcript did not contain *LEU2* but included a part of the *URA3* cistron, which had several in-frame initiation codons and was likely to be translated (Fig. 1G, bottom panel).

Taken together, we successfully screened for rapid functional activation of a prokaryotic-type polycistron in yeast. We found that the translation of cistron 2 is rapidly induced, whereas the activation of cistron 3 required genomic rearrangements eliminating cistron 1, while preserving translation from cistron 2 (now in the first position).

### The activated strain stably translates the bicistronic transcript independent of any *cis-*element

**Figure 2.**
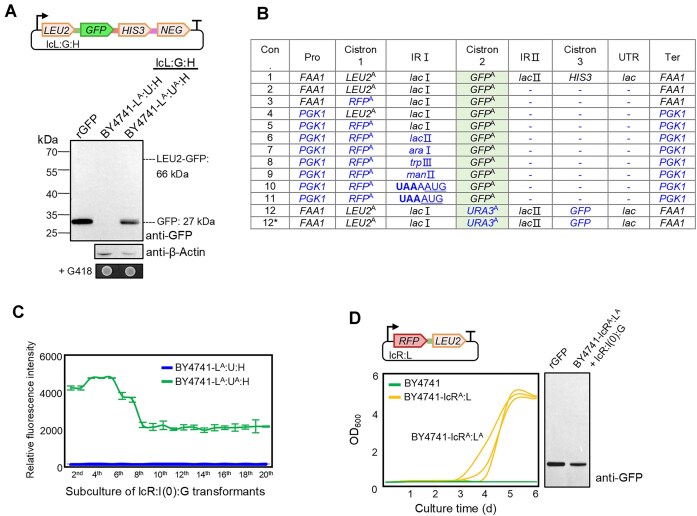
The activated strain stably translates bicistronic transcripts independent of *cis-*elements. (**A**) Cistron 2 is individually translated into a complete protein in the activated strain. The *URA3* reading frame in polycistron L:U:H was replaced with the *GFP* coding region to form polycistron L:G:H, which was introduced into strains BY4741-L^A^:U:H and BY4741-L^A^:U^A^:H as a low-copy plasmid (lcL:G:H). Immunoblotting revealed a single band corresponding to GFP and absence of a Leu2-GFP fusion protein. rGFP: recombinant GFP control. (**B**) Replacement analysis of cistrons and *cis-*elements. The *cis-*elements and cistrons were replaced or deleted based on construct lcL:G:H (Con.1). The resulting constructs were introduced into BY4741-L^A^:U^A^:H and BY4741-L^A^:U^A^:H^A^. Cistron 2 was functional in all constructs (highlighted), whereas function of cistron 3 was not detected in any of them. Con., construct; Pro: promoter; Ter: terminator; IR: intercistronic region, including *lac***I** (51 bp), *lac***II** (65 bp), *ara***I** (10 bp), and*trp***III** (11 bp) from operons of *E. coli*, and *man***II** (14 bp) from *B. subtilis*; **UAA**AAUG indicates just one base A in IR, and **UAA**AUG indicates the lack of an intercistronic region, in that the stop codon (bold) of cistron 1 is directly followed by the start codon (underlined) of cistron 2, as frequently found in prokaryotes; *: construct expressed in BY4741-L^A^:U^A^:H^A^ (all other constructs were expressed in BY4741-L^A^:U^A^:H). (**C**) Once activated, the ability of bicistronic expression persists for at least 20 rounds of subculture in the absence of selection. lcR:I(0):G (construct 11 without intercistronic region) with G418 resistance was introduced into the original strain (BY4741-L^A^:U:H) and the activated strain (BY4741-L^A^:U^A^:H), and the resulting strains were continuously cultivated in complete medium with G418. (**D**) Expression of a plasmid-borne bicistron can be readily induced by selection for another prototrophy. Plasmid lcR:L was introduced into strain BY4741 to yield BY4741-lcR^A^:L. Selection for functional activation of cistron 2 (BY4741-lcR^A^:L^A^) was conducted by incubating cultures of BY4741-lcR^A^:L in -Leu medium. The activation process is represented by the growth curves. Each experiment was conducted in three biological replicates. Due to the lack of a Leu2-specific antibody, lcR:I(0):G was introduced into the activated strain BY4741-lcR^A^:L^A^ to detect the translation of cistron 2 by western blot analysis with an anti-GFP antibody (right panel).

The functional activation of cistron 2 could be explained by activation of its translation as a separate cistron or, alternatively, as fusion with the upstream protein. To distinguish between these possibilities, *GFP* was inserted in the L:U:H polycistron to replace *URA3* (Fig. 2A, upper panel). The resulting low-copy plasmid lcL:G:H was introduced into BY4741-L^A^:U^A^:H and BY4741-L^A^:U:H. Only the activated strain showed GFP fluorescence, and immunoblot analysis revealed a single band corresponding to the size of unfused GFP (Fig. 2A, bottom panel), indicating that cistron 2 was individually translated in the activated strain, consistent with bicistronic expression.

Notably, this gene replacement experiment also demonstrated that bicistronic expression in the activated strain was independent of the coding region. To test if bicistronic expression was dependent on specific *cis-*elements, various elements of the polycistron lcL:G:H were exchanged, including (i) the promoter, (ii) the cistrons, (iii) intercistronic regions, and (iv) the terminator sequence (Fig. 2B). Cistron 2 was faithfully translated into an individual protein in all constructs tested, while cistron 3 did not become functional in any of them (Fig. 2B and Supplementary Fig. S1). These results indicated that the activated strain is capable of translating bicistronic transcripts independent of any *cis-*elements. Even when only a single base separates the stop codon of cistron 1 and the start codon of cistron 2 (**UAA**AAUG), or in the complete absence of an intercistronic region (i.e. the stop codon of cistron 1 being immediately followed by the start codon of cistron 2: **UAA**AUG, a common constellation in prokaryotic operons), the bicistronic transcript was translated in the activated strain.

Notably, the activated strain retained its capacity to translate bicistronic transcripts even after prolonged cultivation without selection pressure. To further confirm this finding, the activated strain was continuously cultivated without selection, and bicistronic expression was maintained even after 20 rounds of subculture (Fig. 2C), indicating that the ability of bicistronic expression, once activated, can persist for a long time.

Taken together, our data show that a bicistron integrated into the yeast genome can be rapidly translationally activated. Next, we examined whether activation can also occur in plasmid-borne cistrons, and upon selection for other traits than uracil prototrophy. To this end, we constructed a bicistronic plasmid with *LEU2* as cistron 2. When this plasmid was introduced into strain BY4741, cistron 2 translation could also be rapidly induced by selection for leucine prototrophy (Fig. 2D), indicating that yeast is capable of activating the translation of bicistronic transcript largely independently of the sequence context.

### Translation of bicistronic transcripts occurs via REI mediated by the 80S ribosome

**Figure 3.**
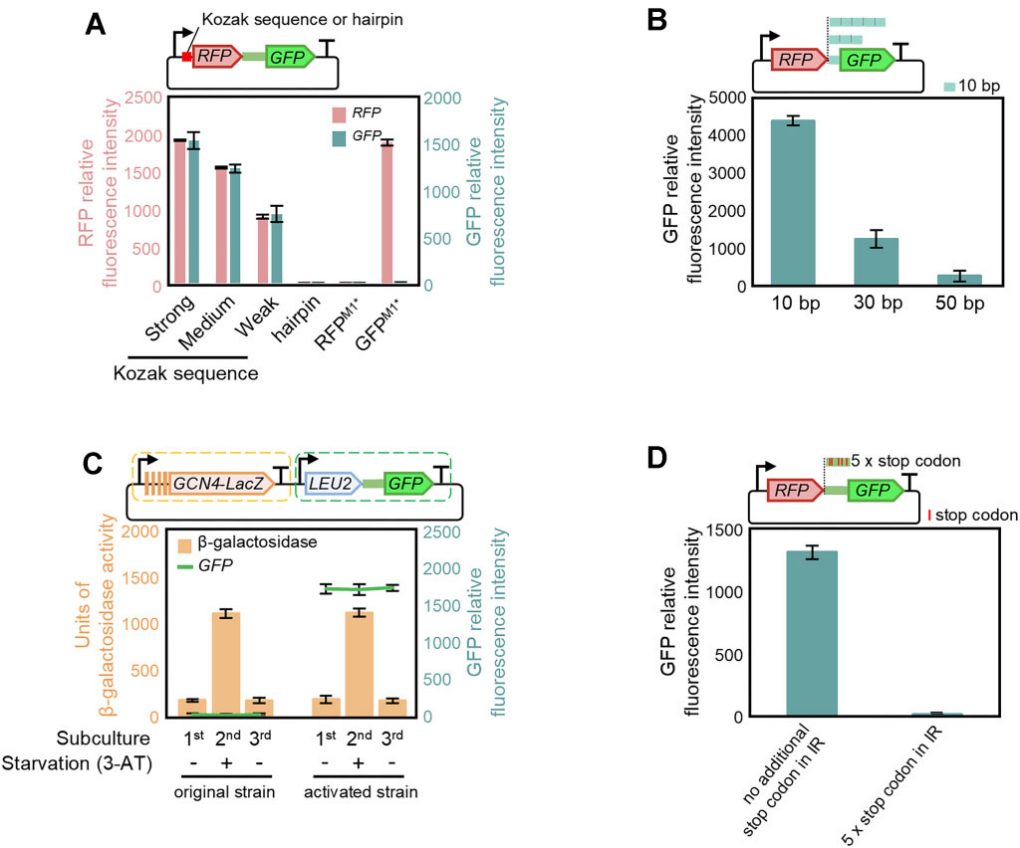
Bicistronic expression is based on REI, which is mediated by the 80S ribosome. (**A**) Correlation between expression of cistron 1 and cistron 2 indicates that cistron 2 translation is mediated by REI. Known *cis-*regulatory elements that control translation initiation, including three different Kozak sequences (Strong: AAAACA; Medium: TAGGTT; Weak: ACGTTC) and a hairpin, were inserted into construct 3 (Fig. 2B) to alter the translation level of cistron 1. For other controls, the start codons of RFP and GFP were respectively mutated to UAA in the construct with strong Kozak sequences. The resulting constructs were introduced into the activated strain BY4741-L^A^:U^A^:H. A highly significant positive correlation of the translation intensity of cistron 2 with that of cistron 1 was observed (*P* < 0.001, *R*^2^ = 0.9994). (**B**) Increased intercistronic spacer length reduces cistron 2 expression. A 10-bp intercistronic region sequence (*ara***I**: GGACACGATA) was inserted in 1, 3, or 5 copies to create intercistronic regions of 10, 30, or 50 bp upstream of cistron 2 in construct lcR:G. Increased intercistronic spacer length reduced the expression of cistron 2. (**C**) Induction of cistron 2 translation does not induce the translation of *GCN4* and vice versa, indicating distinct REI mechanisms. A bicistronic expression cassette and a *GCN4* expression cassette [10] (driven by the *GCN4* promoter and containing four short uORFs fused with *lacZ* as a reporter of *GCN4* expression) were integrated into the same low copy plasmid, and introduced into strains BY4741-L^A^:U:H and BY4741-L^A^:U^A^:H. 10 mM 3-aminotriazole (3-AT) was added to the cultures as a starvation-mimicking inducer for the translation of *GCN4*, which is measured as activity of the β-GAL reporter. (**D**) REI in the activated strain is mediated not by the 40S subunit but by the 80S ribosome. The addition of stop codons in the intercistronic region is expected to inhibit 80S-mediated REI, but not 40S-mediated REI. In addition to the stop codon at the end of RFP, five additional non-consecutive stop codons were introduced into the intergenic region of construct 3 (which contains a 51 bp *lac***I** IR that can effectively reinitiate, Fig. 2B) in the form of point mutations. The resulting construct was then introduced into the activated strain BY4741-L^A^:U^A^:H.

To investigate the mechanism underlying cistron 2 translation, we conducted translational correlation analysis between cistron 1 and cistron 2, in order to distinguish between translation reinitiation, internal initiation and leaky ribosome scanning as potential mechanisms underlying translation of cistron 2. Three Kozak sequences with varying translation initiation efficiencies [4, 38] and a hairpin that does not affect transcription but significantly inhibits translation [39, 40] were used to modify the translation levels of cistron 1. Moreover, nonsense mutations were also introduced at the start codons of cistron 1 or cistron 2 in the construct with strong Kozak sequence to serve as negative controls. The results revealed a highly significant positive correlation (*P* < 0.001, *R*^2^= 0.9994) between the translation intensity of cistron 1 and that of cistron 2 (Fig. 3A), indicating that the translation of cistron 2 strictly depends on cistron 1 translation. Coupling of translation efficiencies is the hallmark of REI.

Classical REI in eukaryotes is mediated by the 40S ribosomal subunit, and typically occurs downstream of short uORFs. A prominent example is the regulation of *GCN4* translation, where the initiation efficiency increases with start codon distance from the stop codon of the upstream short uORF [11, 41–44]. To study the impact of the intercistronic spacer length on REI at cistron 2, intercistronic regions of 10, 30, or 50 bp were tested and their effect on cistron 2 translation in the activated strain was measured. The increased spacer length reduced cistron 2 expression (Fig. 3B). This observation would be compatible with an 80S-mediated REI mechanism, given that the probability of 80S ribosome dissociation increases with the scanning distance [13, 45].

To further rule out conventional 40S-mediated REI, we integrated expression cassettes for *GCN4* [10] and a bicistron into the same plasmid (Fig. 3C), and introduced this plasmid into strains BY4741-L^A^:U:H and BY4741-L^A^:U^A^:H. Application of the starvation-mimicking compound 3-aminotriazole induced *GCN4* expression in both the original and the activated strains but did not induce bicistronic expression in the original strain. Moreover, the basal expression level of *GCN4* did not increase in the activated strain in the absence of starvation induction. The finding that *GCN4* expression levels were very similar in the original strain and the activated strains further indicated that REI in the activated strain was distinct from the 40S-mediated REI typical of *GCN4*. Finally, this result once again showed that the bicistronic expression in the activated strain is stable even after prolonged cultivation, whereas *GCN4* expression strictly depended on induction (Fig. 3C).

Post-termination REI can be mediated by either the 40S ribosomal subunit or the complete 80S ribosome. Given the obvious independence of REI in the activated strain of *cis-*elements or the intercistronic region, which both are essential for 40S-mediated REI, and the observed decrease in cistron 2 expression with increasing intercistronic spacer length, it seems reasonable to assume that REI in the activated strain is mediated by the 80S ribosome. To confirm this conjecture, we introduced five additional stop codons into the intercistronic region. Stop codons are not recognized by 40S subunits, but provide dissociation signals for the 80S ribosome, and, therefore, should only affect 80S-mediated REI [13, 46]. As expected, the five additional stop codons greatly reduced cistron 2 expression (Fig. 3D), thus further supporting an 80S-mediated REI mechanism. Furthermore, given that ribosome recycling is controlled by the termination/pre-recycling complex, the assembly of which is based on stop codon recognition [16, 47], the effect of the additional stop codons suggests that assembly of termination/pre-recycling complex causes REI in the activated strain, rather than the deficiency of complex components such as eRF or Rli1 [14]. Taken together, our data indicated that the activated strain translates bicistronic transcripts via REI, which is mediated not by the 40S subunit but by the 80S ribosome.

### Efficient 80S-mediated REI is evoked by inefficient stop codon recognition

**Figure 4.**
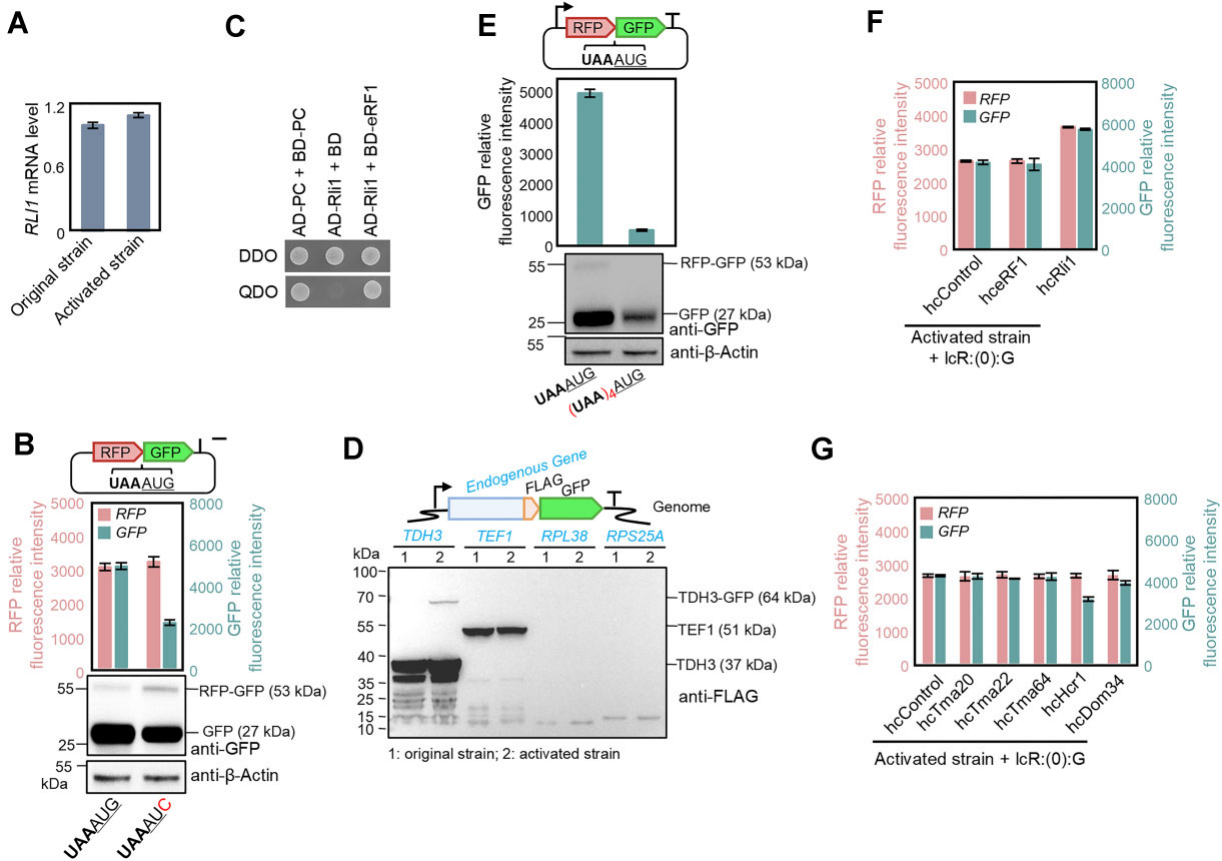
Efficient 80S-mediated REI is caused by mildly defective stop codon recognition. (**A**) *RLI1* expression is not decreased in the activated strain. Quantification of *RLI1* mRNA levels in the original strain (BY4741-L^A^:U:H) and the activated strain (BY4741-L^A^:U^A^:H) by qRT-PCR. (**B**) Start codon selection for REI and bicistronic fusion protein expression in the activated strain. In construct lcR:I(0):G, the AUG start codon of *GFP* was replaced with AUC, and the resulting construct was expressed in strain BY4741-L^A^:U^A^:H. The first in-frame AUG of *GFP* is located 231 bp downstream of the introduced AUC. Immunoblot analysis (bottom panel) shows bands corresponding to the size of GFP and a larger protein corresponding to the size of the RFP–GFP fusion protein. (**C**) Y2H analysis of the interaction between Rli1 and eRF1. DDO, double dropout medium (-Trp/-Leu); QDO, quadruple dropout medium (-Ade/-His/-Trp/-Leu); BD, Gal4 binding domain; AD, Gal4 activation domain; AD-PC + BD-PC, positive control for the interaction. (**D**) Stop-codon readthrough is also observed in the expression of endogenous genes in the activated strain. In the genome of the activated strain (BY4741-L^A^:U^A^:H), *FLAG* tag and *GFP* sequences were inserted at the 3′ end of four randomly selected endogenous genes. Immunoblot analysis detected the expression of two of the genes (*TDH3* and *TEF1*). In the activated strain, Tdh3 additionally accumulated as a fusion protein that likely originates from stop codon readthrough. (**E**) A fortified termination signal after cistron 1 strongly decreases REI and stop codon readthrough. Three additional stop codons were added to the stop codon of cistron 1 to create a stronger termination signal. The resulting constructs were introduced into strains BY4741-L^A^:U:H and BY4741-L^A^:U^A^:H. (**F**) Overexpression of eRF1 or Rli1 does not impair REI, indicating that REI in the activated strain was not evoked by insufficient levels of free eRF1 or Rli1. eRF1 and Rli1 were separately overexpressed from a high-copy plasmid (hceRF1 and hcRli1, respectively) in the activated strain (BY4741L^A^:U^A^:H + lcR:I(0):G). The gene *QDR3*, randomly selected from the genome, served as a control (hcControl). (**G**) Overexpression of 80S ribosome recycling factor Hcr1 reduced REI, further supporting 80S-mediated REI. Tma20, Tma22, Tma64, Hcr1, and Dom34 were separately overexpressed from a high-copy plasmid (hcTma20, hcTma22, hcTma64, hcHcr1, and hcDom34) in the activated strain (BY4741L^A^:U^A^:H + lcR:I(0):G). The control (hcControl) was the same as in panel (F).

Previously reported 80S-mediated REIs include three major types: retroreinitiation [48], REI mediated by P-site tRNA complementarity [49], and REI mediated by Rli1 depletion [13]. However, unlike retroreinitiation [48] and REI mediated by P-site tRNA complementarity [49], the bicistronic expression mechanisms discovered in this study is independent of any specific DNA sequence. Also, the characteristic codons required for these two types of REI are not present within the scannable regions around the stop codon of cistron 1 in any of our constructs. Moreover, both retroreinitiation and REI mediated by P-site tRNA complementarity depend on the presence of an abnormal short upstream ORF, and only REI mediated by Rli1 depletion can occur downstream of a regular reading frame. To test if Rli1 depletion could be involved in the bicistronic expression reported here, we analyzed *RLI1* gene expression in the activated strain and found it to be unaltered (Fig. 4A), indicating that bicistronic REI is not mediated by Rli1 depletion.

Start codon selection is an important feature of efficient translation initiation, but Rli1-mediated REI shows no pronounced preference for the canonical AUG start codon [13]. Our activated strain translated bicistronic transcripts containing different intercistronic regions (from different species and with different sizes) to give rise to cistron 2-encoded proteins of identical size (Supplementary Fig. S1), implying faithful AUG start codon selection. To further investigate the start codon requirements at the second cistron, the AUG of cistron 2 was replaced with AUC or CCC. In the absence of a canonical start codon, reinitiation could still occur from the adjacent reading frames, but cistron 2 expression was significantly decreased (Fig. 4B and Supplementary Fig. S2), indicating that efficient REI in our activated strain depends on the presence of an AUG start codon (start codon preference). Immunoblot analysis unexpectedly revealed accumulation of a fusion protein in the activated strain, its abundance increased as REI at cistron 2 decreased (Fig. 4B). Fusion protein synthesis is caused by stop codon readthrough, which occurs when a near-cognate transfer RNA (tRNA) slips in the A site quicker than the release factor (eRF) [50, 51], leading the continuous peptide elongation rather than peptide release. By contrast, REI is caused by the failure of the ribosome to be dissociated by ribosome dissociation factor (Rli1). To assess the relationship between eRF and Rli1, protein–protein interaction assays in the Y2H system were conducted. These analyses revealed that eRF1 and Rli1 interact (Fig. 4C), consistent with studies that had shown that Rli1 is assembled into the termination/pre-recycling complex via interaction with eRF1 [14, 16, 52]. It has also been demonstrated that the assembly of eRF into the complex is based on stop codon recognition by eRF1 [15, 16, 47, 50]. Our results described above showed that a single stop codon does not provide a sufficiently strong signal for assembly of the termination/pre-recycling complex, but six stop codons do (Fig. 3D). We, therefore, hypothesize that inefficient stop codon recognition reduces the assembly of eRF1 and Rli1 into termination/pre-recycling complexes, thus causing a certain amount of stop codon readthrough and REI in the activated strain.

If stop codon recognition was indeed inefficient, it would also affect endogenous genes, especially for bicistrons. As no functional bicistrons have been reported in yeast, we inserted a *GFP* coding sequence downstream of four randomly selected endogenous genes in the activated strain to form bicistrons to be able to detect stop codon readthrough in endogenous yeast genes. Immunoblot analysis detected the expression of two endogenous genes (*TDH3* and *TEF1*), and a Tdh3–GFP fusion protein, suggesting that stop-codon recognition in endogenous genes is indeed affected in the activated strain (Fig. 4D). The defect in stop-codon recognition in the activated strain was mild, in that it had no obvious effect on the accumulation of the *TDH3* and *TEF1* proteins (Fig. 4D). Intense GFP fluorescence was observed in both the *TDH3:GFP* and the *TEF1:GFP*-containing activated strains (Supplementary Fig. S3), indicating efficient REI on bicistronic transcripts.

To further verify that 80S-mediated REI in the activated strain was evoked by reduced efficiency of stop codon recognition, three additional stop codons were added after the original stop codon of cistron 1 in the bicistronic construct (Fig. 4E) to create a stronger termination signal. Moreover, to exclude potential interference from fusion proteins, a single-base intercistronic region was introduced to ensure the proper separation of two cistrons (Supplementary Fig. S4). As expected, the enhanced stop signal strongly reduced REI and eliminated stop-codon readthrough (as evidenced by loss of the fusion protein; Fig. 4E and Supplementary Fig. S4), further confirming that mildly defective stop codon recognition leads to stop codon readthrough and REI in the activated strain. In addition, the termination signals generated by the different types of stop codons do not affect the amount of fusion protein (Supplementary Fig. S5). In yeast, one possible reason for the reduced efficiency of stop codon recognition is the formation of prions by the translation termination factor eRF3/Sup35 [36]. To verify whether a similar situation occurred in the activated strain, we treated BY4741-L^A^:U^A^:H with guanidine hydrochloride (GdnHCl) to eliminate prions. However, the reinitiation efficiency of the activated strain did not decrease after treatment (Supplementary Fig. S6), suggesting that REI was not caused by prions in the activated strain.

To further confirm that efficient 80S-REI was evoked by inefficient stop codon recognition, we overexpressed release factor eRF1 and 80S-recycling factor Rli1 in the activated strain, as these factors will promote the dissociation of 80S ribosomes, thereby reducing REI [13]. However, elevated levels of eRF1 or Rli1 did not decrease cistron 2 expression. Instead, more Rli1 led to increased cistron 2 expression, which is attributable to enhanced cistron 1 expression (Fig. 4F), given that the expression of the two cistrons is tightly correlated (Fig. 3A). The observed increase in gene expression upon Rli1 overexpression could be due to the roles of Rli1 in facilitating ribosome biogenesis [53, 54] and translation initiation [55]. In agreement with this interpretation, Rli1 overexpression also enhanced the expression of a plasmid-borne gene (Supplementary Fig. S7). We also investigated the role of other ribosome recycling factors: (i) 40S subunit recycling factors Tma20, Tma22, and Tma64 [56]; (ii) 80S recycling factor Hcr1 [57]; and (iii) inactive ribosomes recycling factors Dom34 [58]. Among them, only elevated levels of Hcr1 content led to a significant decrease in the expression level of cistron 2 (Fig. 4G). Hcr1 (also known as eIFj), the component of translation initiation factor 3, has been implicated not only in the recruitment of Rli1 to termination sites facilitating 80S recycling, but also in promoting the loading of release factors (eRF) into the ribosome [56, 57, 59]. These results further support the idea that efficient 80S-mediated REI was responsible for bicistronic expression in the activated strain.

### *NDJ1* and epigenetic modifications are likely involved in the induction process

To further explore the molecular mechanisms underlying the activation of bicistronic mRNA expression and its relationship with stop codon recognition, whole-genome re-sequencing was conducted with the activated strain. Remarkably, no mutations were found in the genome of the activated strain, neither single-nucleotide polymorphisms (SNVs) nor insertions or deletions (indels). To further confirm that there is no genomic mutation in the activated strain, we also conducted Sanger sequencing of repetitive genomic regions, given that mutations in these regions can remain undetected by next-generation re-sequencing methods. In the yeast genome, repetitive sequences are mainly present in the ribosomal DNA (rDNA) region [60–62]. However, we did not find any changes in the rDNA sequences of the activated strain. In line with the very short activation time and the high repeatability of the activation process (Figs 1C and 2D), it stands to reason that yeast has an inherent inducible ability to express bicistronic transcripts.

**Figure 5.**
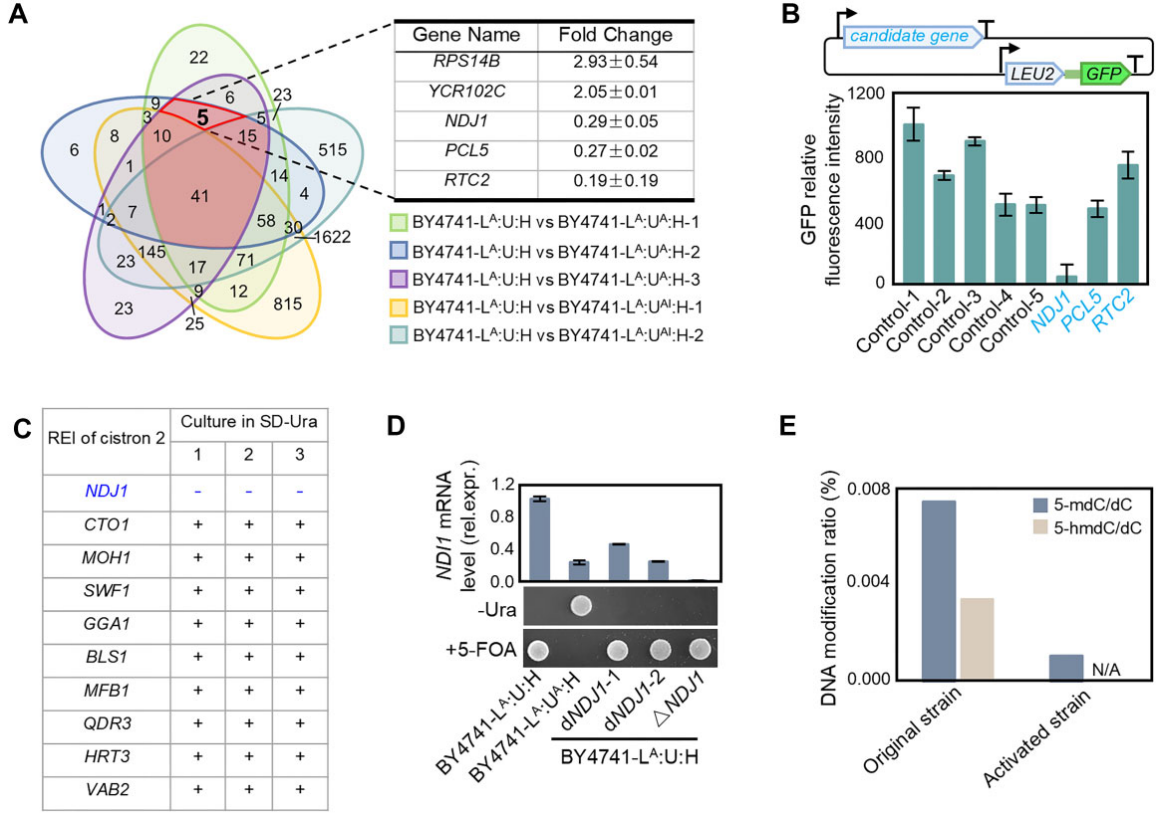
*NDJ1* and epigenetic modifications are involved in the induction process. (**A**) Transcriptome analysis revealed five candidate genes that consistently display differential expression in all activated strains. Activation of bicistronic expression in the activated strain (BY4741-L^A^:U^A^:H) was abrogated through counterselection against *URA3* (cultivation of BY4741-L^A^:U^A^:H on complete medium supplemented with 5-FOA) to generate strain BY4741-L^A^:U^AI^:H (I: inactivation following activation). The Venn diagram shows the significant DEGs across various treatments revealed by transcriptome analysis. The DEGs shared among three independently isolated activated strains (minus the DEGs of BY4741-L^A^:U^AI^:H) were selected as candidate genes (5 genes listed in the table) that potentially are causally involved in the activation of bicistronic expression. (**B**) Enhanced expression of *NDJ1* strongly inhibits REI for bicistronic expression. The downregulated candidate genes were overexpressed in the activated strain together with a reporter cassette for bicistronic expression. Control-1 has only the bicistronic expression detection cassette on the low-copy plasmid. The candidate genes of Control-2 to Control-5 are: *ARO3*, *ADH5*, *RTB5*, and *TMT1* (four randomly-selected endogenous genes). (**C**) Overexpression of *NDJ1* prevents the induction of bicistronic expression. Expression cassettes for *NDJ1* and 9 control genes were inserted into a plasmid with a G418 resistance gene and introduced into strain BY4741-L^A^:U:H to generate a set of original strains. Induction of cistron 2 expression was conducted by incubating cultures in -Ura medium supplemented with G418, with three biological replicates for each culture. In contrast to *NDJ1*, the original strains expressing the 9 randomly selected endogenous control genes all enabled the rapid functional activation of cistron 2. (**D**) Downregulation of *NDJ1* alone is insufficient to induce REI for bicistronic expression. *NDJ1* was knocked down (d*NDJ1*-1 and -2) or knocked out (△*NDJ1*) from the original strain (BY4741-L^A^:U:H). Relative expression levels are shown (top panel). Strains were cultured on medium without uracil or supplemented with 5-FOA to conduct positive or negative selection for REI of *URA3* as cistron 2 (bottom panel). (**E**) Genomic DNA methylation is strongly reduced in the activated strain. Quantification of DNA methylation was conducted by LC-MS/MS in the original strain (BY4741-L^A^:U:H) and activated strain (BY4741-L^A^:U^A^:H). 5-hmdC: 5-hydroxymethyl-2′-deoxycytidine; 5-mdC: 5-methyl-2′-deoxycytidine; dC: deoxycytidine.

Since the rapid induction of inherent functions is primarily regulated at the level of transcription and/or epigenetics [10, 46, 63, 64], we conducted comparative transcriptome analyses in an attempt to identify the upstream factor(s) responsible for bicistronic expression. Transcriptomic data revealed 71 DEGs, marked with red shading (5 + 10 + 15 + 41), in the three independently isolated activated strains compared to the original strain (Fig. 5A). To eliminate genes that are unrelated to the expression phenotype, bicistronic expression was inactivated in BY4741-L^A^:U^A^:H through counterselection against *URA3* to yield strain BY4741-L^A^:U^AI^:H. Transcriptome analysis revealed 66 DEGs shared with BY4741-L^A^:U^A^:H, which were excluded. Finally, 5 DEGs were identified as candidate genes involved in the induction of bicistronic expression, three of which were downregulated [*Nondisjunction 1* (*NDJ1*) [65]; *Pho85 Cyclin 5* (*PCL5*) [66]; *Restriction of Telomere Capping 2* (*RTC2*) [67]], while two were upregulated [(*Ribosomal Protein of the Small subunit 14B* (*RPS14B*) [68]; *Putative quinone oxidoreductase* (*YCR102C*) [69]; Fig. 5A]. Knocking down two upregulated genes (*RPS14B* and *YCR102C*) separately in the activated strain could not disrupt the expression of the bicistron (Supplementary Fig. S8). And compared to controls overexpressing four randomly selected endogenous genes, only overexpression of *NDJ1* led to a significant decrease in cistron 2 expression (Fig. 5B). To further investigate the role of *NDJ1*, we constitutively overexpressed the gene in the original strain to prevent its downregulation. Compared to controls overexpressing nine randomly selected endogenous genes, only *NDJ1* overexpression led to a failure to induce bicistronic expression in stain BY4741-L^A^:U:H (Fig. 5C). Based on this finding, we attempted to install the capacity of bicistronic expression in the original strain BY4741-L^A^:U:H by downregulating or knocking out *NDJ1*. However, neither of these genetic modifications activated cistron 2 (Fig. 5D). Thus, *NDJ1* downregulation is necessary but not sufficient for bicistronic expression.

Our genomic, transcriptomic and proteomic (Supplementary Table S1) analyses also revealed that there were no changes in the DNA sequences or expression levels of genes encoding previously identified REI-related factors such as eRF and Rli1, lending further support to the conclusion that REI was not evoked by changes in the activity of regulatory factors but rather by the assembly of termination/pre-recycling complexes due to inefficient stop-codon recognition.

Given that the expression of some genes significantly changed even though the activation of bicistronic expression was independent of genomic mutations, and the expression capacity was stably maintained for at least 20 subcultures, we considered the possibility that epigenetic regulation is involved in the induction process. We, therefore, quantitatively analyzed differences in DNA and RNA modifications between the original strain and the activated strains. Regarding DNA modifications, both 5-hydroxymethyl-2′-deoxycytidine and 5-methyl-2′-deoxycytidine exhibited a significant decrease in the activated strain (Fig. 5E). At the same time, there have been significant changes in RNA modification (Supplementary Fig. S9). These findings suggest that the epigenetic modifications may be involved in the induction of bicistronic expression.

### Construction of a bicistronic expression strain for biotechnological applications

**Figure 6.**
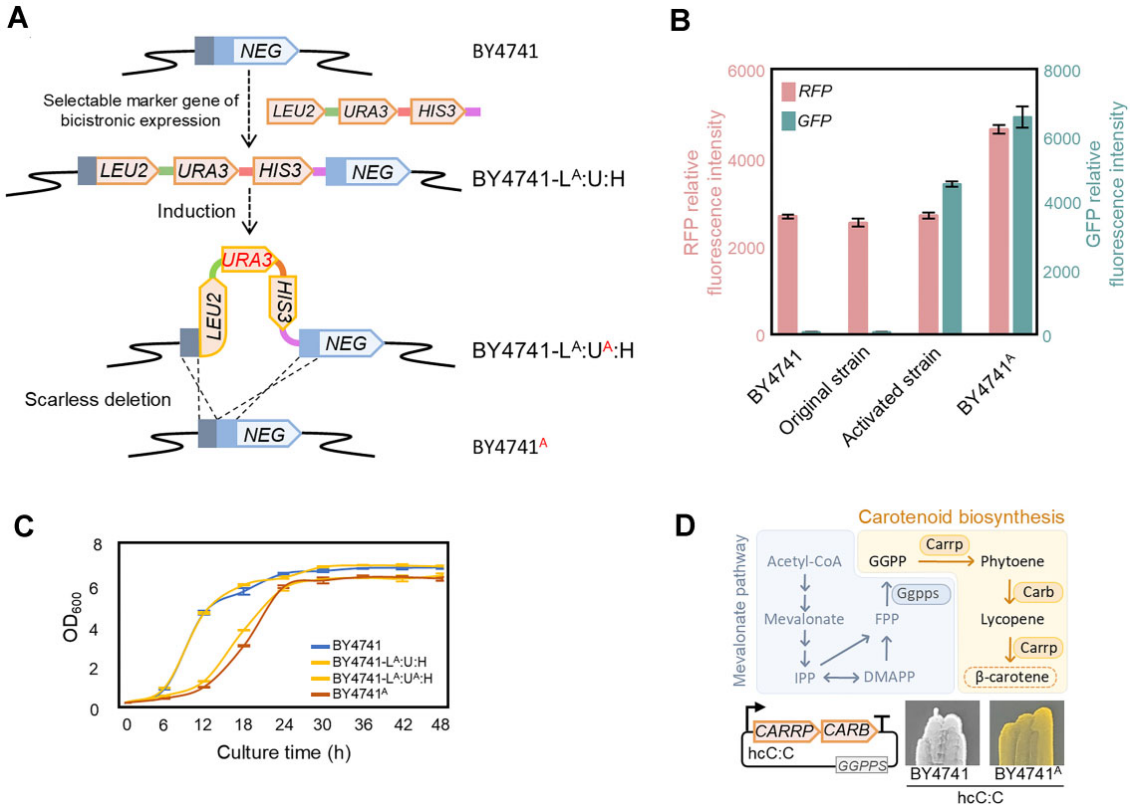
Construction of yeast strain BY4741^A^ for efficient bicistronic expression. (**A**) Flowchart of BY4741^A^ construction. The selectable marker cassette for bicistronic expression (L:U:H) was inserted into the genome of BY4741, and, after activation of bicistronic expression, the marker gene was removed by scarless deletion. The plasmid used for scarless deletion was eliminated through sub-cultivation, and the resulting strain had no sequence changes in its genome compared to the wild-type strain BY4741, as confirmed by PCR and Sanger sequencing. (**B**) BY4741^A^ efficiently expresses a bicistron. Plasmid lcR:I(0):G was introduced into strains BY4741, BY4741-L^A^:U:H, BY4741-L^A^:U^A^:H, and BY4741^A^. Relative fluorescence intensities were measured as proxy of bicistronic expression. (**C**) BY4741^A^ has a longer lag phase but accumulates similar final biomass as the wild-type strain. All strains were cultured in complete medium without selection. (**D**) Application of strain BY4741^A^ for bicistronic expression of functionally related genes to produce a high-value secondary metabolite. Shown is a simplified scheme of the mevalonate pathway of isoprenoid biosynthesis, with the orange background denoting the carotenoid biosynthesis pathway. Ggpps, geranylgeranyl diphosphate synthase; Carrp, bifunctional phytoene synthase and lycopene cyclase; Carb, phytoene desaturase. The carotenoid biosynthesis genes *CARRP* and *CARB* from *Blakeslea trispora* were linked in a synthetic operon (as a spacerless bicistron with **UAA**AUG as stop and start codon) on a high-copy plasmid. Another expression cassette encoding *GGPPS* was added to supply sufficient precursor of carotenoid biosynthesis (GGPP). The construct was introduced into both BY4741 and BY4741^A^. Only strain BY4741^A^ synthesized β-carotene, as evidenced by the pronounced orange phenotype.

To ultimately confirm that the bicistronic expression discovered in this study is stable and independent of genomic mutations, and, at the same time, construct a new yeast strain with the capacity for bicistronic expression, we attempted to develop a BY4741-based strain for the efficient expression of bicistrons. The obtained strong evidence for an epigenetic nature of the expression phenotype (see above, Fig. 5) suggested that such a strain can be developed without any sequence change in the genome. To obtain such a strain (named BY4741^A^), we used the activated strain BY4741-L^A^:U^A^:H that had the wild-type genome except for the insertion of the selectable markers. Consequently, removal of the L:U:H markers by scarless deletion resulted in a strain with the identical genome sequence as the wild-type strain BY4741 (Fig. 6A). Subsequently, construct lcR:I(0):G was introduced into strain BY4741^A^, and it indeed retained the ability to efficiently express bicistrons (Fig. 6B). This experiment conclusively demonstrated that bicistronic expression is an inherent and inducible ability of yeast, independent of exogenous genes or genomic mutations. The growth curves of BY4741^A^ and BY4741-L^A^:U^A^:H were indistinguishable, although they exhibited a longer lag phase compared with BY4741. Nonetheless, the final biomass accumulation reached in BY4741^A^ was very similar to the wild-type strain BY4741 (Fig. 6C).

Prokaryotes utilize operons to co-express functionally related genes, and, in this way, achieve the concerted regulation of biological processes such as biosynthetic pathways. To demonstrate that our new expression strain BY4741^A^ can also utilize bicistrons to co-express biosynthetic pathway genes, the carotenogenic genes *CARB* and *CARRP* from the zygomycete fungus *Blakeslea trispora* were stacked into a bicistron to synthesize the secondary metabolite β-carotene in yeast (Fig. 6D). When this bicistron was introduced into BY4741 and BY4741^A^, only BY4741^A^ exhibited a pronounced orange phenotype indicative of carotenoid synthesis. Thus, the bicistronic expression mechanism uncovered in this study makes the classical eukaryotic model microbe *S. cerevisiae* amenable to the operon-like co-expression of transgenes, thus opening up new opportunities for basic research, biotechnology and synthetic biology.

## Discussion

In the course of this study, we initially confirmed that the bacterial polycistron structure indeed hindered the translation of cistrons 2 and 3 in yeast cells. However, we demonstrated that a polycistron could be rapidly activated in yeast based on the induction of bicistronic expression under selection. The novel bicistronic expression is independent of any *cis-*elements, efficient and stable. Notably, bicistronic expression is mediated by a new type of REI that is mediated by the 80S ribosome and was promoted by inefficient stop codon recognition. The efficiency of 80S-REI decreases with the increase of the intercistronic region, suggesting the 80S ribosome cannot initiate the translation of downstream gene after scanning a longer intercistronic region as effectively as 40S-REI. The induction of bicistronic expression does not require mutations in the genome. Instead, epigenetic modification and downregulation of the *NDJ1* gene are involved. Finally, we developed a yeast strain with the inherent ability to express bicistronic constructs, and successfully employed it to co-express functionally related genes to synthesize a high-value secondary metabolite.

Two major barriers to HGT are (i) the incompatibility of expression elements between donor and recipient such as promoters and translation initiation signals and (ii) fundamental differences in gene structure and transcript processing (e.g. introns impeding eukaryote-to-prokaryote gene transfer and the bacterial operon structure hindering functional prokaryote-to-eukaryote transfer of polycistrons). While the former barrier can be readily overcome by acquisition of regulatory elements from the recipient, the latter is conceivably a much larger obstacle. The activation of polycistrons in eukaryotic recipients seems particularly challenging, given that eukaryotes evolved an mRNA capping-dependent translation initiation mechanism that is generally believed to be causative of eukaryotic organisms having abandoned polycistronic expression. Our study confirmed that yeast does not normally translate the downstream cistrons of a polycistronic mRNA (Fig. 1A and B). However, under conditions where expression of the second cistron confers a selective advantage (e.g. by conferring amino acid prototrophy), the ability to express bicistrons can be rapidly activated (Figs 1C and 2D). Although we did not detect a translational activation event of cistron 3 in our experimental design, the 80S-REI mechanism of activation is not likely to be cistron 2-specific and we could not rule out the possibility of polycistronic expression activation under special conditions.

**Figure 7.**
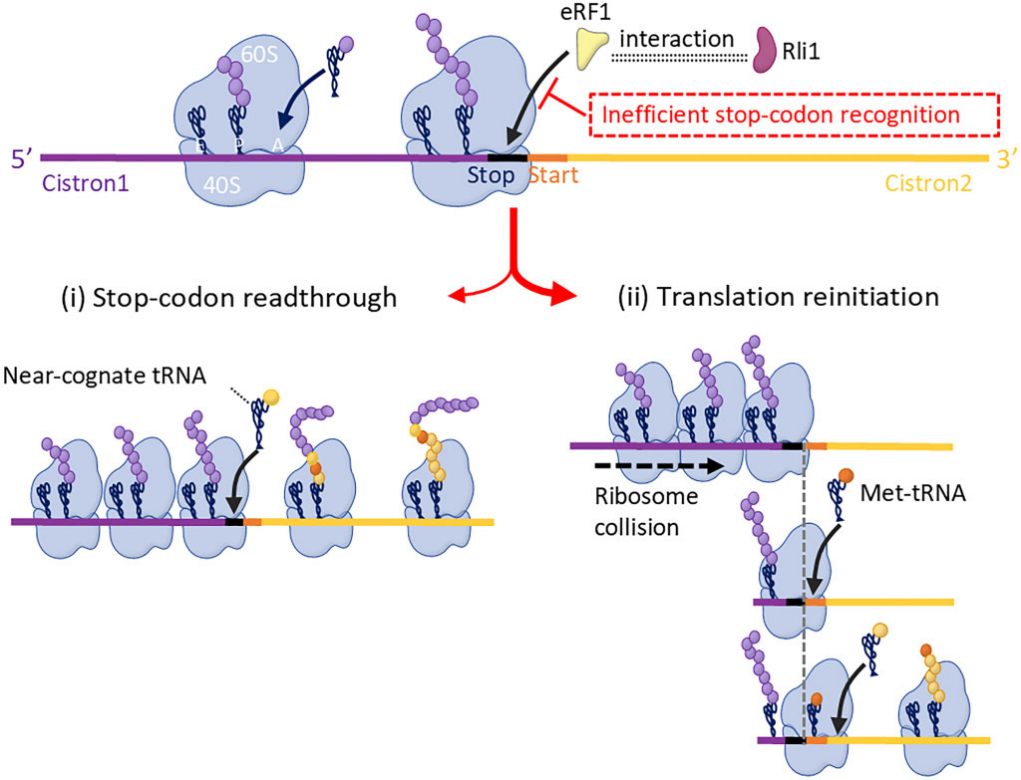
Schematic model depicting the fate of 80S ribosomes upon inefficient stop codon recognition in the activated strain. Inefficient stop codon recognition leads to a fraction of eRF1 that cannot assembled into termination/pre-recycling complexes to initiate peptide release, Rli1 assembly and ribosome dissociation. Consequently, a small proportion of ribosomes (with unreleased nascent polypeptide chains) will pause at the stop codon. The paused ribosomes can have two fates: (i) a small proportion may be decoded by a near-cognate tRNA, thus resulting in stop codon readthrough, whereas (ii) the majority of the paused ribosomes will be pushed forward due to collision with upstream ribosomes, thus bypassing the stop codon and triggering peptide release. These ribosomes can then reinitiate translation (REI).

During the normal translation termination and ribosome recycling process in eukaryotes, eRF1 recognizes the stop codon and releases the nascent polypeptide from the ribosome. Subsequently, Rli1 dissociates the 60S ribosomal subunit from the 80S ribosome. Shortage of eRF1 will cause stop codon readthrough [50, 52], whereas shortage of Rli1 will led to REI [13]. Consequently, REI caused by Rli1 depletion is not accompanied by stop codon readthrough [13]. However, we observed a correlation between REI and stop codon readthrough in bicistronic expression (Fig. 4B–E), along with assembly of Rli1 into termination/pre-recycling complexes (based on Rli1 interaction with eRF, Fig. 4C) [14, 52]. These findings indicated that the REI discovered in this study is not simply due to Rli1 depletion but is mediated by the concerted peptide release (by eRF1) and ribosome recycling (by Rli1). We also found that, in the activated strain, stop codon recognition by eRF1 is inefficient (Figs 3D and 4E), and stop codon recognition is required for assembly of eRF into the termination/pre-recycling complex. This finding suggests that a fraction of eRF may not have been assembled into termination/pre-recycling complexes to initiate peptide release, Rli1 assembly and ribosome dissociation, and therefore, some ribosomes with unreleased peptides pause at the stop codon. The paused ribosomes have two fates (Fig. 7): (i) a small proportion can incorporate a near-cognate tRNA, resulting in stop-codon readthrough, whereas (ii) the majority of the paused ribosomes collide with upstream ribosomes, thus bypassing the stop codon and triggering peptide release. These ribosomes can then reinitiate translation (REI) [10, 13, 16]. In agreement with this model, readthrough increased when REI decreased, as evidenced by start codon mutagenesis (Fig. 4B). It seems reasonable to assume that the reduced efficiency of translation initiation caused by removal of the canonical start codon AUG increases the residence time of ribosomes in the termination–reinitiation region, thus causing even more subsequent ribosomes to stall at the stop codon, in turn providing more opportunities for readthrough. Taking all data together, we concluded that the activation of bicistronic expression in yeast is due to a new type of REI, which is inducible by selection, mediated by the 80S ribosome and promoted by mildly defective stop codon recognition.

In this study, stop codon readthrough (due to inefficient stop-codon recognition) is induced by selection for prototrophy, which involves starvation during the selection period. Stop codon readthrough has been reported to be enhanced by starvation in other eukaryotic cells. For example, serum starvation enhances nonsense mutation readthrough in mammalian cells [70, 71]. Although the exact mechanism underlying the induction by starvation is currently unclear, the apparent involvement of epigenetic modifications and the requirement for downregulation of *NDJ1* observed in this study suggest a two-stage process. First, epigenetic changes induced by the starvation-related selection process (Fig. 5E) cause changes in the transcriptome (Fig. 5A). Subsequently, changes in the expression of a subset of relevant genes such as the downregulation of *NDJ1* (Fig. 5B–D) cause reduced efficiency of stop-codon recognition, possibly by factors competing with eRF [50]. *NDJ1* is currently annotated as a telomere-associated meiotic protein [72]. Although not obviously related to the translation termination/pre-recycling process, the annotated function as nucleic acid-associated protein would be compatible with a dual function or a moonlighting function as RNA-associated protein affecting stop codon recognition. In addition to Ndj1, several other factors may be involved in the regulation of bicistronic expression: (i) the other unidentified DEGs in the transcriptome; (ii) RNA modifications, as there are a large number of changes in RNA modifications in the activated strain (Supplementary Fig. S9). These modifications may alter the expression of the genes regulating translation termination and initiation, thereby participating in the regulation of bicistronic expression. However, the detailed mechanism by which Ndj1 influences stop codon recognition remains to be elucidated.

It is conceivable that the biological role of the activation of bicistronic expression is to enhance phenotypic variation and, in this way, increase flexibility in the response to challenging environmental conditions. The capacity to express downstream cistrons cannot only facilitate the rapid activation of horizontally transferred polycistrons of bacterial origin, but can also greatly enhance the diversity of the proteome to improve fitness under stressful conditions. In summary, our data reported here demonstrate that yeast has the cryptic ability to translate bicistronic mRNAs, and this ability is rapidly inducible by selection to improve fitness.

## Supplementary Material

gkaf220_Supplemental_Files

## Data Availability

The NGS datasets of transcriptome generated and analyzed in this study are available from NCBI under the accession number BioProject PRJNA1155626.
