# Supplementary material for: A hidden intrinsic ability of bicistronic expression based on a novel translation reinitiation mechanism in yeast
Source: Nucleic Acids Res. 2025 Mar 28;53(6):gkaf220. doi: 10.1093/nar/gkaf220 (PMC11952965; doi:10.1093/nar/gkaf220)
Supplement: gkaf220_Supplemental_Files [file gkaf220_supplemental_files.zip › Supplementary Data.pdf]

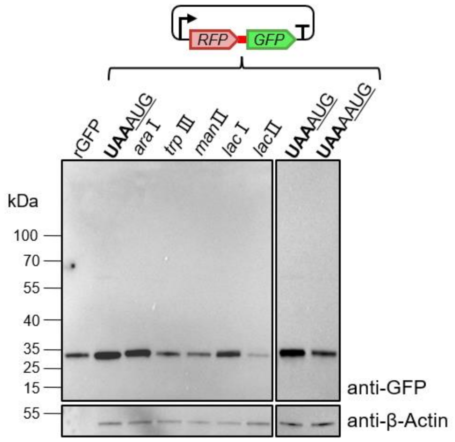

Figure S1. Immunoblot analysis of the translation products of cistron 2 with different intercistronic regions in the activated strain. The activated strains (BY4741-L^{A}:U^{A}:H) containing the constructs 5-11 with the different intercistronic regions (Figure 2B) were used for western blot analysis. The first lane contains a recombinant GFP protein (rGFP) as control.

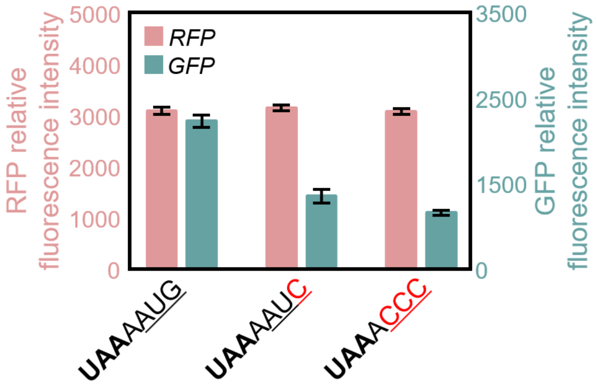

Figure S2. The apparent start codon preference for REI was revealed in the activated strain. In lcR:I(1):G, the AUG of GFP was replaced with AUC or CCC to destroy the start codon, the resulting construct was expressed in BY4741-L^{A}:U^{A}:H. Red indicates the replacement. After the replacement with AUC, the first in-frame AUG of *GFP* located 231-bp downstream of AUC or CCC.

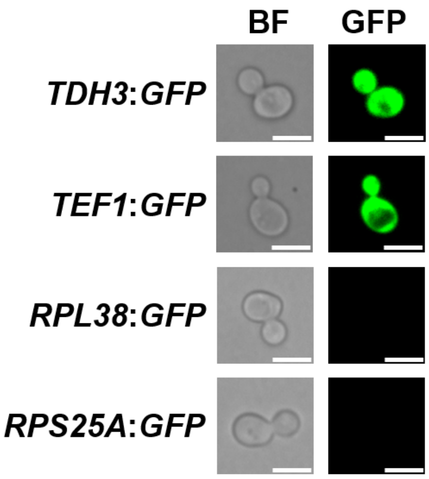

Figure S3. Intense GFP fluorescence was observed in both the *TDH3*:*GFP* and the *TEF1*:*GFP*-containing activated strains. The scale bars for the images are 5 μm. BF, Bright field.

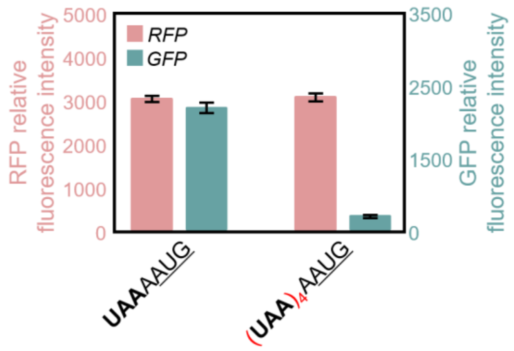

Figure S4. Stronger stop signal for Cistron1 could dramatically decrease REI. Another three successive stop codons were added to the stop codon of Cistron1 of lcR:I(1):G to create a stronger stop signal. The resulting constructs were introduced BY4741-L^{A}:U:H and BY4741-L^{A}:U^{A}:H.

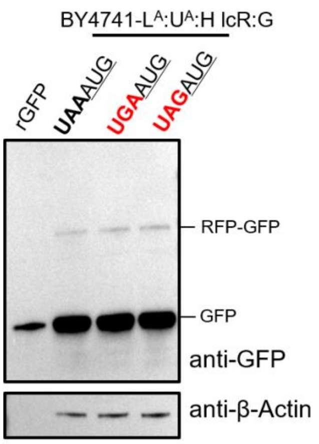

Figure S5. Different types of stop codons will not affect the production of fusion proteins. In lcR:I(0):G, the stop codons UAA of *RFP* was replaced with UGA or UAG, the resulting construct was expressed in BY4741-L^A:U^A:H. Red indicates the replacement.

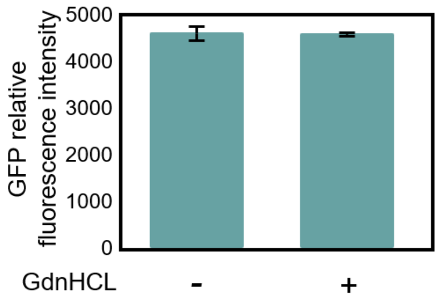

Figure S6. REI was not caused by prions in the activated strain. BY4741-L^A:U^A:H lcR:I(0):G were treated (+) or not treated (-) with 4mM guanidine hydrochloride (GdnHCL) to eliminate prions. The REI efficiency in the activated strain did not decrease after treatment.

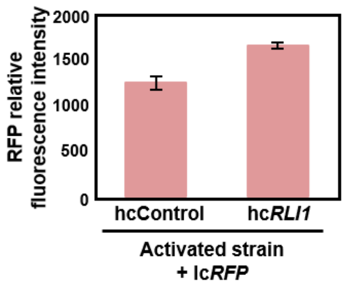

Figure S7. Overexpression of Rli1 enhanced the expression of *RFP* in lc*RFP*. *RFP* expression was driven by the *FAA1* promoter from a low-copy plasmid with G418 resistance (lc*RFP*). The gene *QDR3*, randomly selected from the genome, was inserted into the overexpression vector as a control (hcControl). Then, hcControl/hc*RLI1* were introduced into the activated strain (BY4741L^{A}:U^{A}:H + lcR:I(0):G).

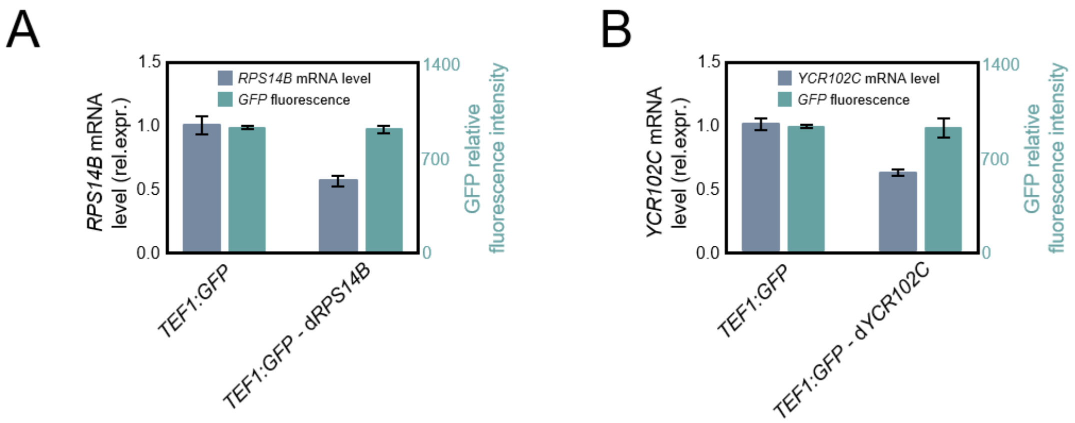

Figure S8. Down-regulation of 2 up-regulated genes could not inhibited REI for bicistronic expression.

(A) Down-regulation of *RPS14B* could not disrupt REI for bicistronic expression. *RPS14B* was knocked down (*TEF1:GFP*-d*RPS14B*) from BY4741-L^{A}:U^{A}:H *TEF1:GFP*.

(B) Down-regulation of *YCR102C* could not disrupt REI for bicistronic expression. *YCR102C* was knocked down (*TEF1:GFP*-d*YCR102C*) from BY4741-L^{A}:U^{A}:H *TEF1:GFP*.

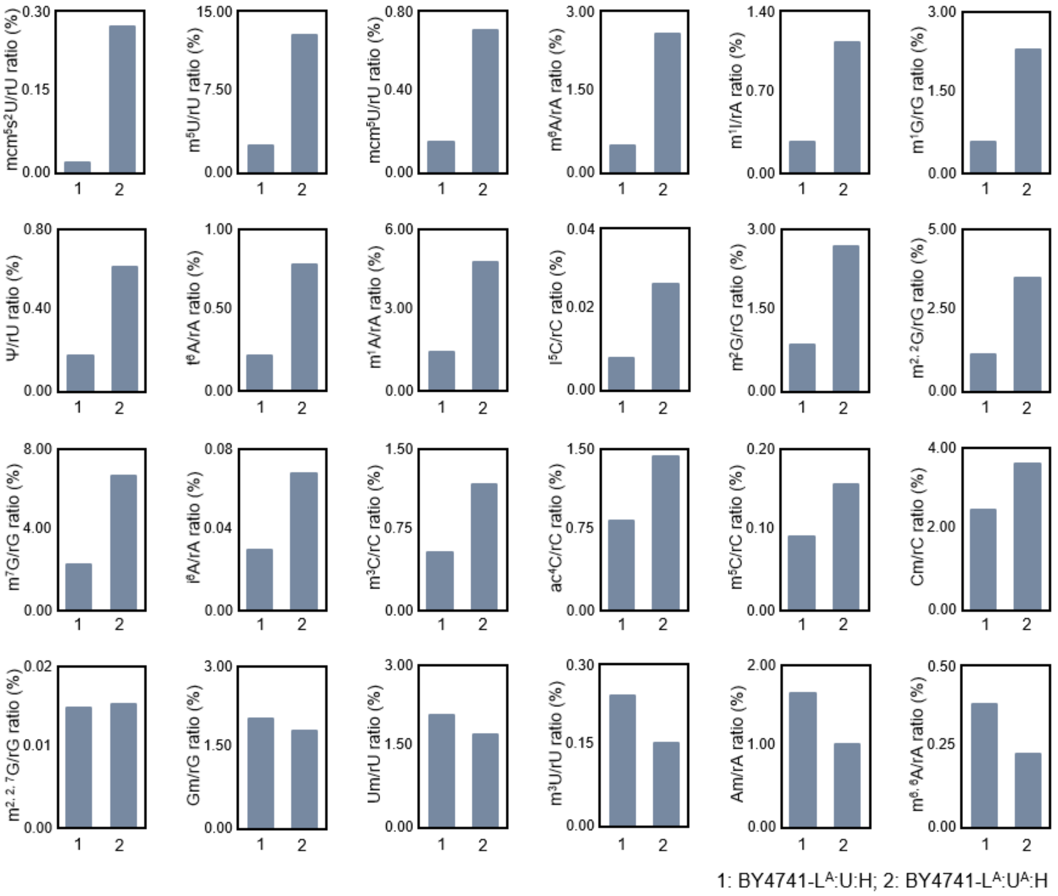

Figure S9 RNA modifications was dramatically increased in activated strain. Quantification of the RNA modifications was detected by LC-MS/MS. Twenty-four RNA modifications were detected, with eighteen showing an increase in BY4741-L^A:U^A:H, five showing a decrease, and one showing no difference. mcm^5s^2U: 5-methoxycarbonylmethyl-2-thiouridine; m^5U: 5-methyluridine; mcm^5U: 5-methoxycarbonylmethyluridine; m^6A: N6-methyladenosine; m^1I: 1-methylinosine; m^1G: 1-methylguanosine; Ψ: pseudouridine; t^6A: N6-threonylcarbamoyladenosine; m^1A: 1-methyladenosine; I^5C: 5-iodocytidine; m^2G: N2-methylguanosine; $m^{2,\ 2}G$: N2,N2-dimethylguanosine; m^7G: 7-methylguanosine; i^6A: N6-isopentenyladenosine; m^3C: 3-methylcytidine; ac^4C: N4-acetylcytidine; m^5C: 5-methylcytidine; Cm: 2'-O-methylcytidine; $m^{2,\ 2,\ 7}G$: N2,N2,7-trimethylguanosine; Gm: 2'-O-methylguanosine; Um: 2'-O-methyluridine; m^3U: 3-methyluridine: Am: 2'-O-methyladenosine; $m^{6,\ 6}A$: N6,N6-dimethyladenosine; rG: guanosine; rC: cytidine; rU: uridine; rA: adenosine.

Table S2. List of oligonucleotides used in this study.

Primers for the generation of hybridization probes

Gene	Forward primer (5'-3')	Reverse primer (5'-3')
LEU2	GTAACTTTGCATCCGACTCTCTT	AGCGGCCATTCTTGTGATTC
URA3	ATGGAGGGCACAGTTAAGC	GCCTGCTTCAAACCGCTA
HIS3	CCCCTAGCGATAGAGCACTC	CTTCAGTGGTGTGATGGTCG

Primers for the 5’ RACE

Gene	Reverse primer (5'-3')
HIS3-1	CTTCAGTGGTGTGATGGTCGTCTATGTG
HIS3-2	CATATGGTCCAGAAACCCTATACCTGTG
HIS3-3	GGGAAGATCGAGTGCTCTATCGCT

Primers for real-time qRT-PCR

Gene	Forward primer (5'-3')	Reverse primer (5'-3')
ACT1	GGATTCTGAGGTTGCTGCTTT	TGACCCATACCGACCATGATAC
RLI1	GTAACTTTGCATCCGACTCTCTT	AGCGGCCATTCTTGTGATTC
NDJ1	ATGGAGGGCACAGTTAAGC	GCCTGCTTCAAACCGCTA
PRS14B	GCCAGAATCTACGCCTCCTT	ATTCGTCTCTGTCGGCCTTG
YCR102C	GTTGCTGGTAACCCGACTGA	TTTGACAATTTGGCCGGCAG

Oligonucleotide sequences for sgRNAs

Name	Sequence (5'-3')
FAA1 CRISPR sgRNA	GCATACGGGACAGCAGAGAT
URA3(M1*) CRISPR sgRNA	ATATGTAGCTTTCGATTAAA
HIS3 CRISPR sgRNA	GGATGAGGCACTTTCCAGAG
HIS3(M1*) CRISPR sgRNA	ATCACTCCGTTATGATATGT
TDH3 CRISPR sgRNA	AAGTAAATTCACTTAAGCCT
TEF1 CRISPR sgRNA	CAAAAGGCTGCTAAGAAATA
RPL38 CRISPR sgRNA	AACAGATTATAAGAAAAATA
RPS25A CRISPR sgRNA	GCTTCTGAATAAACAGGGAA
PRS14B CRISPRi sgRNA	GCCTTCTACGAGTATAAAAG
YCR102C CRISPRi sgRNA	TCTAACCATATTCTATAAAA
NDJ1 CRISPR sgRNA	CAAAACCTACAAGCTAATTC
NDJ1 CRISPRi sgRNA-1	TTCGTACTCAGTGACGTACC
NDJ1 CRISPRi sgRNA-2	CAGCCTGTAGCATCATCCTC
L:U:H CRISPR sgRNA	GAAGAGTAAAAAATTGTACT
