# Supplementary material for: A hidden intrinsic ability of bicistronic expression based on a novel translation reinitiation mechanism in yeast
Source: Nucleic Acids Res. 2025 Mar 28;53(6):gkaf220. doi: 10.1093/nar/gkaf220 (PMC11952965; doi:10.1093/nar/gkaf220)
Supplement: gkaf220_Supplemental_Files [file gkaf220_supplemental_files.zip › Table S1. Differential proteins in the proteome-1..pdf]

Table S1. Differential proteins in the proteome.

Only in the original strain	Only in the activated strain
YPR167C MET16	YLR055C SPT8
YPR155C NCA2	YGL150C INO80
YPR140W TAZ1	YOR018W ROD1
YPR070W MED1	YGR245C SDA1
YPR051W MAK3	YDR243C PRP28
YPR042C PUF2	YGL143C MRF1
YPR026W ATH1	YMR102C LAF1
YPL223C GRE1	YER013W PRP22
YPL186C UIP4	YLR287C YLR287C
YPL171C OYE3	YMR067C UBX4
YPL118W MRP51	YOR207C RET1
YPL116W HOS3	YDL019C OSH2
YPL110C GDE1	YPL132W COX11
YPL104W MSD1	YJR050W ISY1
YPL070W MUK1	YOR281C PLP2
YPL003W ULA1	YPR055W SEC8
YOR385W YOR385W	YJL063C MRPL8
YOR373W NUD1	YCL059C KRR1
YOR304W ISW2	YLR195C NMT1
YOR288C MPD1	YGL241W KAP114
YOR267C HRK1	YPL175W SPT14
YOR244W ESA1	YBR111W-A SUS1
YOR227W HER1	YDR083W RRP8
YOR179C SYC1	YBR146W MRPS9
YOR167C RPS28A	YDR297W SUR2
YOR138C RUP1	YJL085W EXO70
YOR129C AFI1	YAL017W PSK1
YOR115C TRS33	YDL092W SRP14
YOR109W INP53	YLR034C SMF3
YOR083W WHI5	YOR196C LIP5
YOR075W UFE1	YOR119C RIO1
YOR057W SGT1	YJL033W HCA4
YOR020W-A MCO10	YNL022C RCM1
YOR008C SLG1	YDR060W MAK21
YOL154W ZPS1	YMR047C NUP116
YOL126C MDH2	YDR279W RNH202
YOL025W LAG2	YBR200W BEM1
YNR058W BIO3	YDR383C NKP1
YNR057C BIO4	YNR049C MSO1
YNR007C ATG3	YCR008W SAT4
YNL284C-B YNL284C-B	YHR187W IKI1
YNL278W CAF120	YPL115C BEM3
YNL277W MET2	YKL114C APN1
YNL202W SPS19	YBR142W MAK5
YNL192W CHS1	YPL224C MMT2
YNL167C SKO1	YNR074C AIF1
YNL097C PHO23	YDR507C GIN4
YMR267W PPA2	YGL185C YGL185C
YMR262W YMR262W	YDR240C SNU56

YMR255W GFD1
YMR234W RNH1
YMR219W ESC1
YMR194C-B CMC4
YMR160W CVM1
YMR107W SPG4
YMR054W STV1
YMR016C SOK2
YML117W NAB6
YML080W DUS1
YML075C HMG1
YML058W SML1
YML054C CYB2
YML052W SUR7
YML050W AIM32
YML015C TAF11
YLR423C ATG17
YLR396C VPS33
YLR393W ATP10
YLR387C REH1
YLR345W YLR345W
YLR327C TMA10
YLR324W PEX30
YLR299W ECM38
YLR293C GSP1
YLR292C SEC72
YLR284C ECI1
YLR181C VTA1
YLR177W YLR177W
YLR128W DCN1
YLR114C AVL9
YLR107W REX3
YLR082C SRL2
YLR072W LAM6
YLR056W ERG3
YLR006C SSK1
YLL062C MHT1
YLL057C JLP1
YKR100C SKG1
YKR071C DRE2
YKR069W MET1
YKR013W PRY2
YKR009C FOX2
YKL194C MST1
YKL129C MYO3
YKL092C BUD2
YKL091C YKL091C
YKL079W SMY1
YKL063C YKL063C
YKL048C ELM1
YKL038W RGT1
YHR196W UTP9
YNR032C-A HUB1
YGL061C DUO1
YNL075W IMP4
YLR045C STU2
YDR295C HDA2
YDR088C SLU7
YJR093C FIP1
YJR138W IML1
YJL010C NOP9
YKL143W LTV1
YDR444W YDR444W
YBR130C SHE3
YER123W YCK3
YLR108C YLR108C
YER127W LCP5
YDL213C NOP6
YPR169W JIP5
YLR131C ACE2
YDL030W PRP9
YLR321C SFH1
YDR481C PHO8
YHR122W CIA2
YMR158W MRPS8
YNL267W PIK1
YDL203C ACK1
YLR144C ACF2
YOR336W KRE5
YPL093W NOG1
YOL144W NOP8
YJL204C RCY1
YNL141W AAH1
YPL043W NOP4
YOR056C NOB1
YJR092W BUD4
YGL111W NSA1
YML029W USA1
YIL104C SHQ1
YKR093W PTR2
YPR091C NVJ2
YIL153W RRD1
YKR038C KAE1
YDR108W TRS85
YHL007C STE20
YLL036C PRP19
YHR168W MTG2
YGR200C ELP2
YMR167W MLH1
YBL069W AST1
YML102W CAC2
YCL031C RRP7

YKL008C LAC1
YKL001C MET14
YJR154W YJR154W
YJR137C MET5
YJR130C STR2
YJR102C VPS25
YJR091C JSN1
YJR062C NTA1
YJR024C MDE1
YJR001W AVT1
YJL168C SET2
YJL153C INO1
YJL016W TPH3
YJL005W CYR1
YIR039C YPS6
YIR017C MET28
YIR001C SGN1
YIL162W SUC2
YIL160C POT1
YIL088C AVT7
YIL047C SYG1
YIL045W PIG2
YHR178W STB5
YHR176W FMO1
YHR171W ATG7
YHR045W DDE1
YHL019C APM2
YHL003C LAG1
YGR286C BIO2
YGR251W NOP19
YGR165W MRPS35
YGR157W CHO2
YGR152C RSR1
YGR138C TPO2
YGR133W PEX4
YGR106C VOA1
YGR032W GSC2
YGL244W RTF1
YGL237C HAP2
YGL232W TAN1
YGL196W DSD1
YGL180W ATG1
YGL174W BUD13
YGL095C VPS45
YGL006W PMC1
YFR030W MET10
YFR017C IGD1
YFR015C GSY1
YFR011C MIC19
YFL002W-A YFL002W-A
YER175C TMT1
YNL176C TDA7
YER176W ECM32
YNR027W BUD17
YJL197W UBP12
YDL148C NOP14
YGL220W BOL2
YGR235C MIC26
YKL189W HYM1
YIL035C CKA1
YFL026W STE2
YFL024C EPL1
YPR141C KAR3
YCR057C PWP2
YDL150W RPC53
YLL008W DRS1
YGR271C-A EFG1
YCR047C BUD23
YKL205W LOS1
YAL041W CDC24
YGR113W DAM1
YPL270W MDL2
YLR015W BRE2
YCR020W-B HTL1
YLR386W VAC14
YHR088W RPF1
YDR200C VPS64
YBR273C UBX7
YPR112C MRD1
YDR449C UTP6
YGR252W GCN5
YNL254C RTC4
YNL020C ARK1
YJR142W YJR142W
YBR030W RKM3
YML013W UBX2
YNL233W BNI4
YNL223W ATG4
YDR051C DET1
YCR016W RBP95
YNL002C RLP7
YMR212C EFR3
YKR028W SAP190
YHR186C KOG1
YOR320C GNT1
YGR058W PEF1
YGR127W YGR127W
YMR043W MCM1
YLR022C SDO1
YDR237W MRPL7
YBR058C UBP14
YBR192W RIM2

YER166W DNF1
YER040W GLN3
YER019C-A SBH2
YER015W FAA2
YEL012W UBC8
YDR530C APA2
YDR518W EUG1
YDR515W SLF1
YDR490C PKH1
YDR475C JIP4
YDR464W SPP41
YDR462W MRPL28
YDR460W TFB3
YDR439W LRS4
YDR404C RPB7
YDR400W URH1
YDR389W SAC7
YDR313C PIB1
YDR305C HNT2
YDR301W CFT1
YDR288W NSE3
YDR229W IVY1
YDR216W ADR1
YDR207C UME6
YDR182W CDC1
YDR179C CSN9
YDR164C SEC1
YDR116C MRPL1
YDR096W GIS1
YDR081C PDC2
YDR055W PST1
YDL238C GUD1
YDL198C GGC1
YDL102W POL3
YDL095W PMT1
YDL090C RAM1
YDL076C RXT3
YDL044C MTF2
YCR091W KIN82
YCR068W ATG15
YCR059C YIH1
YCR027C RHB1
YCR021C HSP30
YCR020C-A MAK31
YBR283C SSH1
YBR281C DUG2
YBR262C MIC12
YBR251W MRPS5
YBR229C ROT2
YBR213W MET8
YBR208C DUR12
YOL077W-A ATP19
YGL129C RSM23
YNL085W MKT1
YHL009C YAP3
YPR029C APL4
YHR006W STP2
YPL217C BMS1
YNR026C SEC12
YDR398W UTP5
YER026C CHO1
YCR072C RSA4
YGR006W PRP18
YPL006W NCR1
YHR072W ERG7
YFR041C ERJ5
YLR002C NOC3
YPL153C RAD53
YBR193C MED8
YML114C TAF8
YJR131W MNS1
YOR145C PNO1
YLR405W DUS4
YNL317W PFS2
YDR390C UBA2
YOR174W MED4
YHR060W VMA22
YHR041C SRB2
YKL005C BYE1
YPL023C MET12
YOR195W SLK19
YGR241C YAP1802
YKR024C DBP7
YNL225C CNM67
YGR128C UTP8
YLR223C IFH1
YLL035W GRC3
YHR062C RPP1
YGR099W TEL2
YML046W PRP39
YMR210W MGL2
YDR080W VPS41
YOR118W RTC5
YGL210W YPT32
YKL052C ASK1
YBR289W SNF5
YLL018C-A COX19
YPL266W DIM1
YAR018C KIN3
YGL198W YIP4
YKR025W RPC37
YLL034C RIX7

YBR173C UMP1
YBR151W APD1
YBR145W ADH5
YBR135W CKS1
YBR081C SPT7
YBR062C YBR062C
YBR046C ZTA1
YBR023C CHS3
YBR005W RCR1
YBL103C RTG3
YBL029C-A YBL029C-A
YAR027W UIP3
YAL062W GDH3
YAL048C GEM1
YAL039C CYC3
YAL023C PMT2
Q0045 COX1

YNL212W VID27
YMR288W HSH155
YOR016C ERP4
YER126C NSA2
YAL025C MAK16
YBL067C UBP13
YDR264C AKR1
YCR024C SLM5
YKL021C MAK11
YDL156W CMR1
YNL136W EAF7
YOR211C MGM1
YHR207C SET5
YMR289W ABZ2
YOL115W PAP2
YHR034C PIH1
YMR171C EAR1
YGR223C HSV2
YMR228W MTF1
YFL030W AGX1
YAL033W POP5
YLR417W VPS36
YDR311W TFB1
YOR229W WTM2
YLR413W INA1
YMR239C RNT1
YBR002C RER2
YPR190C RPC82
YPL042C SSN3
YKL173W SNU114
YOR316C COT1
YGR117C YGR117C
YPL015C HST2
YDL170W UGA3
YGR093W DRN1
YER168C CCA1
YOR252W TMA16
YMR184W ADD37
YNL297C MON2
YGR012W MCY1
YPL195W APL5
YPL095C EEB1
YJL203W PRP21
YKL149C DBR1
YBR065C ECM2
YPR023C EAF3
YOR081C TGL5
YMR273C ZDS1
YGR072W UPF3
YNL164C IBD2
YJL109C UTP10

YER047C SAP1
YLL006W MMM1
YOR091W TMA46
YPR161C SGV1
YGL017W ATE1
YPR048W TAH18
YHL038C CBP2
YJL061W NUP82
YIR033W MGA2
YHR086W NAM8
YDR324C UTP4
YKL051W SFK1
YDR156W RPA14
YGR283C UPA1
YLR119W SRN2
YPR143W RRP15
YBR246W RRT2
YBR107C IML3
YGL238W CSE1
YJL141C YAK1
YAL014C SYN8
YDR356W SPC110
YDL112W TRM3
YNR023W SNF12
YKR070W YKR070W
YAL044W-A BOL1
YJL122W ALB1
YPL263C KEL3
YER005W YND1
YLR265C NEJ1
YGR007W ECT1
YFL007W BLM10
YDR036C EHD3
YDR337W MRPS28
YHL014C YLF2
YLR137W RKM5
YPR144C NOC4
YPR137W RRP9
YIL079C AIR1
YKL116C PRR1
YLR226W BUR2
YNL027W CRZ1
YNL224C SQS1
YBR287W YBR287W
YEL043W GTA1
YJL039C NUP192
YKR037C SPC34
YMR193W MRPL24
YER154W OXA1
YGL233W SEC15
YGL086W MAD1

YKL074C MUD2
YJL098W SAP185
YLR021W IRC25
YDR376W ARH1
YER002W NOP16
YGL071W AFT1
YMR223W UBP8
YDL004W ATP16
YIL007C NAS2
YFL008W SMC1
YKL130C SHE2
YNL252C MRPL17
YOL033W MSE1
YPL112C PEX25
YDR496C PUF6
YOR266W PNT1
YOR188W MSB1
YBR125C PTC4
YHR115C DMA1
YGL151W NUT1
YML006C GIS4
YMR061W RNA14
YCR054C CTR86
YKR036C CAF4
YGL159W YGL159W
YDR531W CAB1
YLR239C LIP2
YML130C ERO1
YJR084W YJR084W
YOL129W VPS68
YMR112C MED11
YOR064C YNG1
YNL091W NST1
YCL029C BIK1
YJL125C GCD14
YPR097W LEC1
YGL145W TIP20
YMR052W FAR3
YGR081C SLX9
YHR098C SFB3
YLR019W PSR2
YJL011C RPC17
YLR310C CDC25
YDR508C GNP1
YJL112W MDV1
YKR029C SET3
YPL020C ULP1
YDR424C DYN2
YLL011W SOF1
YDR017C KCS1
YOL124C TRM11

YOR048C RAT1
YLR409C UTP21
YNR047W FPK1
YDL001W RMD1
YKL075C AAN1
YBR123C TFC1
YDR146C SWI5
YMR177W MMT1
YHR111W UBA4
YLR207W HRD3
YKL095W YJU2
YBR176W ECM31
YCR003W MRPL32
YHL004W MRP4
YOL068C HST1
YKL087C CYT2
YJL030W MAD2
YNR015W SMM1
YBL102W SFT2
YDR268W MSW1
YDR485C VPS72
YHR167W THP2
YGR156W PTI1
YBR236C ABD1
YKR055W RHO4
YOR098C NUP1
YNL152W INN1
YOR275C RIM20
YOR329C SCD5
YGL066W SGF73
YNL263C YIF1
YHR066W SSF1
YLR272C YCS4
YDR465C RMT2
YPL194W DDC1
YGL016W KAP122
YPR025C CCL1
YCL016C DCC1
YLR066W SPC3
YER061C CEM1
YOR226C ISU2
YML119W YML119W
YMR049C ERB1
YOL145C CTR9
YIL131C FKH1
YDR435C PPM1
YOL135C MED7
YAR014C BUD14
YNR052C POP2
YOR085W OST3
YOR001W RRP6

YNL047C SLM2
YMR192W GYL1
YOR360C PDE2
YDR303C RSC3
YBL085W BOI1
YBR141C BMT2
YER016W BIM1
YDL056W MBP1
YLR096W KIN2
YOR144C ELG1
YPR125W YLH47
YDL070W BDF2
YFL002C SPB4
YIL084C SDS3
YBL105C PKC1
YNL298W CLA4
YDL018C ERP3
YOL108C INO4
YGL065C ALG2
YPL140C MKK2
YMR188C MRPS17
YEL017W GTT3
YML103C NUP188
YOR357C SNX3
YOL141W PPM2
YLR129W DIP2
YMR144W FDO1
YNL023C FAP1
YJL049W CHM7
YIL123W SIM1
YDL003W MCD1
YGR143W SKN1
YPL183C RTT10
YDR077W SED1
YJR032W CPR7
YMR224C MRE11
YNL292W PUS4
YDR169C STB3
YFR005C SAD1
YLR238W FAR10
YIL173W VTH1
YKL106W AAT1
YLR243W GPN3
YPL103C FMP30
YDR041W RSM10
YPR180W AOS1
YJR006W POL31
YBL034C STU1
YNL272C SEC2
YJL074C SMC3
YOR104W PIN2

YMR172W HOT1
YDL175C AIR2
YKR079C TRZ1
YBL033C RIB1
YNL072W RNH201
YIL107C PFK26
YLR074C BUD20
YDR097C MSH6
YDR448W ADA2
YKL017C HCS1
YBR119W MUD1
YER139C RTR1
YOR143C THI80
YJL069C UTP18
YCL052C PBN1
YPL101W ELP4
YHR046C INM1
YGL169W SUA5
YER142C MAG1
YIL091C UTP25
YHR169W DBP8
YGR060W ERG25
YNL110C NOP15
YPR176C BET2
YPR178W PRP4
YOL080C REX4
YNR022C MRPL50
YPL259C APM1
YDR110W FOB1
YPR113W PIS1
YIL127C RRT14
YIL096C BMT5
YKR089C TGL4
YLR212C TUB4
YKL191W DPH2
YKR087C OMA1
YDR100W TVP15
YJR046W TAH11
YER088C DOT6
YOR094W ARF3
YLR063W BMT6
YLR306W UBC12
YML121W GTR1
YLR095C IOC2
YOL062C APM4
YOR026W BUB3
YLR372W ELO3
YCL054W SPB1
YPL139C UME1
YLR332W MID2
YHR003C TCD1

YPR063C YPR063C
YGL091C NBP35
YDL202W MRPL11
YGR247W CPD1
YIL004C BET1
YER050C RSM18
YJL126W NIT2
YAL015C NTG1
YCR036W RBK1
YJL044C GYP6
YPR174C CSA1
YJR003C MRX12
YBR188C NTC20
YDR056C EMC10
YLR020C YEH2
YPR045C THP3
YDR425W SNX41
YIL003W CFD1
YGL224C SDT1
YDR323C PEP7
YGL073W HSF1
YHR058C MED6
YGR276C RNH70
YCR087C-A YCR087C-A
YHR056C RSC30
YML071C COG8
YNL329C PEX6
YOR233W KIN4
YNL070W TOM7
YOR017W PET127
YOR052C TMC1
YMR240C CUS1
YGR145W ENP2
YNL062C GCD10
YJL133W MRS3
YIR007W EGH1
YDR325W YCG1
YLR375W STP3
YKR068C BET3
YOR103C OST2
YBR061C TRM7
YDL167C NRP1
YDR061W YDR061W
YCL014W BUD3
YIL148W RPL40A
YNL119W NCS2
YNL215W IES2
YPR175W DPB2
YKL065C YET1
YLR005W SSL1
YIL008W URM1

YMR042W ARG80
YNR038W DBP6
YLR287C-A RPS30A
YNL169C PSD1
YER034W YER034W
YNL310C ZIM17
YLL014W EMC6
YHR206W SKN7
YLR363C NMD4
